# Convenient method for resolving degeneracies due to symmetry of the magnetic susceptibility tensor and its application to pseudo contact shift-based protein–protein complex structure determination

**DOI:** 10.1007/s10858-012-9623-8

**Published:** 2012-04-10

**Authors:** Yoshihiro Kobashigawa, Tomohide Saio, Masahiro Ushio, Mitsuhiro Sekiguchi, Masashi Yokochi, Kenji Ogura, Fuyuhiko Inagaki

**Affiliations:** 1 Department of Structural Biology, Faculty of Advanced Life Science, Hokkaido University, N-21, W-11, Kita-ku, Sapporo, 001-0021 Japan; 2 Graduate School of Life Science, Hokkaido University, Sapporo, Japan; 3 Analysis and Pharmacokinetics Research Labs, Department of Drug Discovery, Astellas Pharma Inc., Tokyo, Japan

**Keywords:** Differential scanning fluorometry, Pseudo contact shift, Lanthanide binding tag, FKBP12, FRB, mTOR

## Abstract

Pseudo contact shifts (PCSs) induced by paramagnetic lanthanide ions fixed in a protein frame provide long-range distance and angular information, and are valuable for the structure determination of protein–protein and protein–ligand complexes. We have been developing a lanthanide-binding peptide tag (hereafter LBT) anchored at two points via a peptide bond and a disulfide bond to the target proteins. However, the magnetic susceptibility tensor displays symmetry, which can cause multiple degenerated solutions in a structure calculation based solely on PCSs. Here we show a convenient method for resolving this degeneracy by changing the spacer length between the LBT and target protein. We applied this approach to PCS-based rigid body docking between the FKBP12-rapamycin complex and the mTOR FRB domain, and demonstrated that degeneracy could be resolved using the PCS restraints obtained from two-point anchored LBT with two different spacer lengths. The present strategy will markedly increase the usefulness of two-point anchored LBT for protein complex structure determination.

**Electronic supplementary material:**

The online version of this article (doi:10.1007/s10858-012-9623-8) contains supplementary material, which is available to authorized users.

## Introduction

Long-range distance and angular information is useful for the structural analysis of large proteins, multidomain proteins and protein complexes (Gaponenko et al. 2002, 2004; Battiste and Wagner 2000; Vlasie et al. 2007; Tang and Clore 2006; Rumpel et al. 2007; Bertini et al. 2009). Paramagnetic lanthanide ions induce several NMR effects on observed nuclei, including pseudo-contact shifts (PCSs) and residual dipolar couplings (RDCs) due to anisotropy of the magnetic susceptibility tensor (Δχ-tensor; Bertini et al. 2005, 2008; Otting 2008). PCSs provide distance and angular information between the lanthanide ion and the observed nuclei situated up to approximately 40 Å from the lanthanide ion (Allegrozzi et al. 2000), whereas RDCs provide molecular alignment information independent of distance (Bertini et al. 2001; Barbieri et al. 2002). Therefore, paramagnetic lanthanide ions are useful probes for solution structure determination by NMR and have been applied successfully to metalloproteins (Bertini et al. 2001, 2004, 2007; Barbieri et al. 2002; Pintacuda et al. 2006, 2007; Allegrozzi et al. 2000). Metal ions such as Ca^2+^ and Mg^2+^ ions can be replaced by lanthanide ions as they share similar chemical properties. However, these approaches are limited, in principle, to metalloproteins.

For the application of paramagnetic lanthanide probes to non-metalloproteins, a wide variety of lanthanide-anchoring tags has been developed, including lanthanide binding peptide tags (Su et al. 2006, 2008a; Gaponenko et al. 2000; Wohnert et al. 2003; Martin et al. 2007; Ma and Opella 2000; Zhuang et al. 2008) and synthetic lanthanide chelating reagents (Dvoretsky et al. 2002; Haberz et al. 2006; Pintacuda et al. 2004; Prudencio et al. 2004; Rodriguez-Castaneda et al. 2006; Ikegami et al. 2004; Leonov et al. 2005; Gaponenko et al. 2002, 2004; Vlasie et al. 2007; Keizers et al. 2007, 2008; Su et al. 2008b; Swarbrick et al. 2011a, b; Graham et al. 2011). These tags are attached to the target proteins through N- or C-terminal fusion (Gaponenko et al. 2000; Wohnert et al. 2003; Martin et al. 2007; Ma and Opella 2000; Zhuang et al. 2008), insertion into the loop region (Barthelmes et al. 2011) or the formation of a disulfide bond with cysteine residues (Su et al. 2006, 2008a, b; Dvoretsky et al. 2002; Haberz et al. 2006; Pintacuda et al. 2004; Prudencio et al. 2004; Ikegami et al. 2004; Leonov et al. 2005; Gaponenko et al. 2002, 2004; Vlasie et al. 2007; Keizers et al. 2007, 2008; Swarbrick et al. 2011a, b; Graham et al. 2011). However, the mobility of the tag relative to the target protein reduces the anisotropic paramagnetic effect (Bertini et al. 2004, 2007; Su et al. 2008a). Hence, rigidity of the tag relative to the target protein is necessary for obtaining quantitative structural information using paramagnetic lanthanide probes.

The two-point anchoring method affords a promising approach to the rigid fixation of the lanthanide binding tag to the target protein. The symmetrically designed synthetic chelators can be anchored to the protein via two disulfide bonds (Keizers et al. 2007, 2008). Most of these tags, however, are not commercially available at present. Recently, we reported a method that utilizes a lanthanide-binding peptide tag, CYVDTNNDGAYEGDEL (LBT; Nitz et al. 2003, 2004; Su et al. 2006, 2008a), linked to the target protein via two anchoring points, a disulfide bridge and an N-terminal fusion (Saio et al. 2009). This two-point anchored LBT has one advantage for protein NMR research in that it can be expressed as a fusion protein with the target protein using *E. coli*. This method was first applied to the B1 immunoglobulin binding domain of protein G (GB1) as a model protein to evaluate the Δχ-tensor of the paramagnetic lanthanide ion (Saio et al. 2009). We then applied two-point anchored LBT to the PCS-based structure determination of protein–protein complexes (Saio et al. 2010), drug screening, and structure determination of drug–protein complexes (Saio et al. 2011). However, the magnetic susceptibility tensor has symmetry, and thus gives eight degenerate solutions in a structure determination based solely on PCS restraints (Saio et al. 2010). This degeneracy cannot be fully resolved by the combined use of multiple PCS data sets derived from several lanthanide ions. In order to overcome this degeneracy, it is crucial to obtain another PCS data set that possesses a different orientation of the principal axes and a different position of the paramagnetic center relative to the target protein. Several sets of the data are available by introducing the tag into different positions on the target protein. In many cases, however, the identification of additional fixation points for tagging is not straightforward.

Here we show that the direction of the principal axes of the Δχ-tensor and the metal position relative to the target protein can be conveniently modulated by modifying the spacer length between LBT and the target protein. This was confirmed for three proteins, the GB1, FKBP12 and Grb2 SH2 domains. Moreover, we applied this approach to the PCS-based rigid body docking between the FKBP12-rapamycin complex and the mTOR FRB domain, and demonstrated that the degeneracy could be resolved by using PCS restraints obtained from two LBT-attached constructs of different spacer lengths with three and four amino acid residues. The present study will markedly increase the usefulness of the two-point anchored LBT for protein complex structure determination.

## Materials and methods

### Construction of the expression plasmid

The fragment encoding the FRB domain (2015–2114) of human mTOR (GenBank ID of AAA58486) and the fragment encoding full-length human FKBP12 (GenBank ID of AAA35844) were cloned into pGBHPS (Kobashigawa et al. 2009). For construction of the expression vector of LBT-attached FKBP12, the fragment encoding FKBP12 (2–107) was cloned into pGTL (Saio et al. 2010; Supplementary Fig. 1). The expression vectors for LBT-attached GB1 and Grb2 SH2 were constructed as described previously (Saio et al. 2009, 2011).

### Protein expression and purification

Two-point anchored LBT-attached Grb2 SH2 and GB1 were prepared as described previously (Saio et al. 2009, 2011). FKBP12, two-point anchored LBT-attached FKBP12, and the FRB domain of mTOR were prepared as follows. Proteins were expressed at 25 °C in *E. coli* strain Rossetta2 (DE3). For the unlabeled samples, cells were grown in Luria–Bertani media. For the uniformly ^15^N- or ^13^C/^15^N-labeled samples, cells were grown in M9 media containing ^15^NH_4_Cl (2 g/L), Celtone-N powder (0.2 g/L) (Cambridge Isotope Laboratories, USA) and unlabeled glucose (10 g/L), or ^15^NH_4_Cl (2 g/L), Celtone-CN powder (0.2 g/L) (Cambridge Isotope Laboratories, USA) and [U–^13^C] glucose (4 g/L), respectively. We also prepared an inversely labeled sample under a ^15^N background. For preparation of the inversely labeled sample, 1 g/L of non-labeled amino acid (A, H, K, M, R or W) or a combination of non-labeled amino acids (F/Y, L/V or A/F/H/I/K/L/M/R/V/W/Y) was added. Considering both the previous results of inversely amino acid selective-labeling (Krishnarjuna et al. 2010) and amino acid biosynthesis pathway of *E. coli* (Waugh 1996), we selected single or combination of amino acids each of which was assumed to exhibit low isotope scrambling. Consistent with the previous results (Krishnarjuna et al. 2010), signal intensity of Ile was reduced by isotope scrambling in L/V inversely amino acid selective-labeled sample. FKBP12 without two-point anchored LBT was purified using Ni–NTA resin (Qiagen) affinity chromatography, followed by tag removal by HRV3C protease and gel filtration using Superdex75 (GE Healthcare). LBT-attached FKBP12 was purified by affinity chromatography using glutathione Sepharose 4B (GE Healthcare), followed by gel-filtration using Superdex75 (GE Healthcare). After gel-filtration, LBT-attached FKBP12 was incubated with 1 mM 5, 5′-ditiobis(2-nitrobenzoic acid) (DTNB) for 2 h at 4 °C to link the N-terminal Cys of LBT and the Cys on the target proteins via an intramolecular disulfide bond (Saio et al. 2009). The reaction was performed under low FKBP12 concentration ranging from 10 to 20 μM. Rapamycin was added to the FKBP12 sample before starting the oxidization reaction. The oxidized two-point anchored LBT-attached proteins were further purified by gel-filtration chromatography on a Superdex 75 (GE Healthcare). The FRB domain of mTOR was expressed in the inclusion body, and retrieved by high-pressure refolding (Schoner et al. 2005; Qoronfleh et al. 2007; Saio et al. 2010). Details of the refolding process will be published elsewhere.

### Differential scanning fluorometry

A real-time PCR device (Mx3005p, Stratagene) was used to monitor protein unfolding by tracking increase in the fluorescence intensity of the fluorophore SYPRO Orange (Sigma). SYPRO Orange was diluted to 1:60 with 20 mM MES buffer (pH 7.0) containing 150 mM NaCl, and used as stock solution for DSF measurement. SYPRO Orange stock solution (2.5 μL) was mixed with 22.5 μL of the L1- to L5-FKBP12-rapamycin complex (10 μM) containing one equivalent molar of LuCl_3_ in 20 mM MES and 150 mM NaCl (pH 7.0). They were incubated in optical cap sample tubes (strips of 8; Agilent Technologies) in the RT-PCR device. The samples were heated at 1 °C per min, from 35 to 95 °C. After base-line correction, the unfolding fractions were estimated and plotted against temperature.

### NMR spectroscopy

Samples were prepared in 20 mM MES buffer (pH 7.0) with 150 mM NaCl for FKBP12-rapamycin, FRB-rapamycin and FKBP12-rapamycin-FRB, in 20 mM MES buffer (pH 6.5) with 50 mM NaCl for the GB1, and in 20 mM Tris buffer (pH 7.2) with 100 mM NaCl for the Grb2 SH2 domain. For the assignment of the ^1^H, ^15^N and ^13^C resonances of FKBP12-rapamycin, FRB-rapamycin and FKBP12-rapamycin-FRB, a standard set of heteronuclear NMR spectra were recorded using Protein Pack pulse sequences (Varian, Inc., Palo Alto, CA, USA). For the assignment of FRB in the FKBP12-rapamycin-FRB ternary complex, ^1^H–^15^N HSQC spectra of seven inversely amino acid selective-labeled samples (A, H, K, M, R, W, F/Y or L/V) were used for amino acid type determination and confirmation of the assignment. For assignment of the PCS peaks of FKBP12-rapamycin, the ^1^H–^15^N HSQC spectrum of an eleven amino acid (A/F/H/I/K/L/M/R/V/W/Y) inversely labeled sample was also used to reduce spectral overlap. The signal assignments and PCS assignments for GB1 and Grb2 SH2 were conducted as described previously (Saio et al. 2009, 2011). All NMR experiments were performed on Inova 800, 600 or 500 MHz NMR spectrometers (Varian, USA) at 25 °C. Spectra were processed using the NMRPipe program (Delaglio et al. 1995) and data analysis was performed with the help of the Olivia program developed in our laboratory (Yokochi et al. http://fermi.pharm.hokudai.ac.jp/olivia/).

### Tensor calculation

The Δχ-tensors for the FKBP12-rapamycin complex, GB1 and Grb2 SH2 domains were calculated from the PCS values and the structure of the FKBP12-rapamycin complex (Van Duyne et al. 1991, 1fkb.pdb), and the GB1 (Saio et al. 2009, 2rpv.pdb) and Grb2 SH2 domains (Ogura et al. 2008, 1x0n.pdb) based on Eq. (1) using the Numbat program (Schmitz et al. 2008),

1 $$ \Updelta \delta^{\text{pcs}} = \frac{1}{{12\pi r^{3} }}\left[ {\Updelta \chi_{{{\text{ax}} }} \left( {3\cos^{2} \theta - 1} \right) + \frac{3}{2}\Updelta \chi_{{{\text{rh}} }} \sin^{2} \theta \cos 2\phi } \right] $$

where Δδ^PCS^ is the pseudo contact shift, *r*, θ and ϕ are the polar coordinates of the nucleus with respect to the principal axis of the magnetic susceptibility tensor, and Δχ_ax_ and Δχ_rh_ are the axial and rhombic components of the magnetic susceptibility tensor.

### Docking

PCS-based rigid body docking was carried out using the Xplor-NIH program (Schwieters et al. 2003, 2006), equipped with PARA restraints for Xplor-NIH (Banci et al. 2004). At the start of the docking calculation, the relative orientation and position of the FRB domain were randomized to generate 100 starting structures that were located within 100 Å from the FKBP12. The coordinates of the metal were fixed at the positions which were determined by Δχ-tensor-fits from the PCSs observed for the L3-FKBP12-rapamycin and L4-FKBP12-rapamycin complexes. The FRB domain moiety from the FKBP12-rapamycin-FRB ternary complex (Liang et al. 1999, 1fap.pdb) was used for docking studies. We used the coordinates of the FKBP12 moiety in this complex for rigid body docking studies, since we determined the Δχ-tensor values for FKBP12 using FKBP12-rapamycin binary complex (Van Duyne et al. 1991, 1fkb.pdb). As the structures of the rapamycin moieties in the binary and ternary complexes differ from each other in the FRB binding region, which could cause a steric crash with the FRB domain in the rigid-body docking calculation, therefore we omitted the rapamycin moiety during the docking calculation. The rigid body docking calculation was performed based on the PCS restraints. During the calculation, the coordinates of FKBP12 and the metal were fixed, whereas those of FRB were freely rotated and translated. For the PCS restraints, pseudo atoms representing the Δχ-tensor axes were introduced. The atom representing the origin of the axis was restrained within 0.02 Å of the metal, while the coordinates of the Δχ-tensor were freely rotated around the origin. The target function was calculated based on two terms: the least square energy penalty for PCS restraints (E_PCS_; Banci et al. 2004), and a quartic van der Waals repulsion term (E_repel_). Ö radius scale factor was decreased from 1.0 to 0.78. The Xplor-NIH script for the docking calculation is provided as Supporting Information.

## Results

### Design and differential scanning fluorometry analysis of two-point anchored LBT-attached FKBP12

**Fig. 1:**
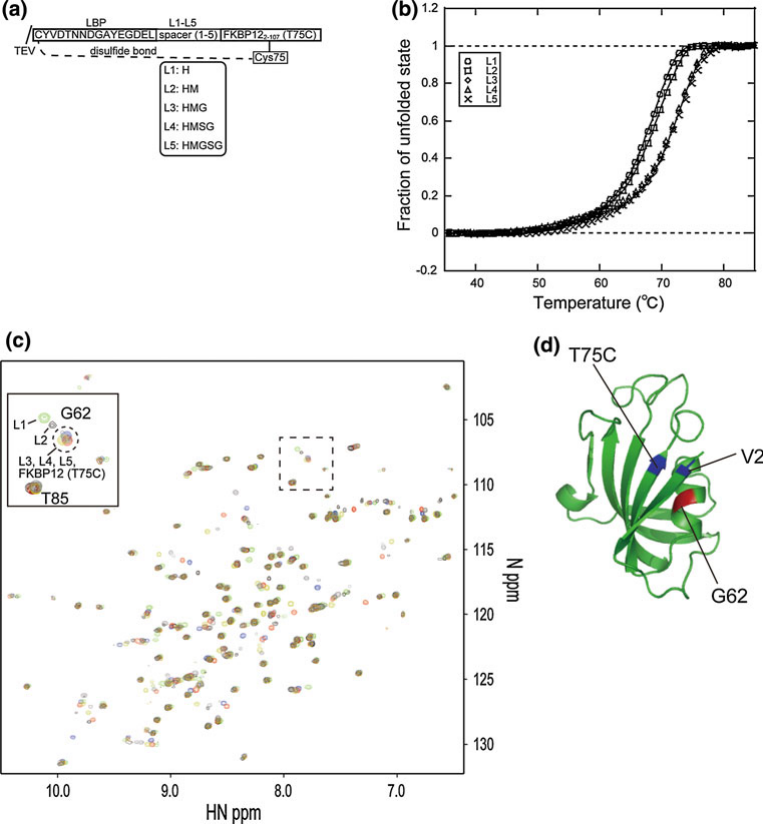
**a** Schematic representation of the two-point anchored LBT-attached FKBP12 construct. The spacer sequence is enclosed in the *box*. **b** The thermal unfolding curve of two-point anchored LBT-attached FKBP12 in the presence of one equivalent molar Lu^3+^. **c** Overlay of the ^1^H–^15^N HSQC spectra of FKBP12 (T75C) without the two-point anchored LBT (*blue*), and L1- (*green*), L2- (*black*), L3- (*red*), L4- (*dark yellow*) and L5-FKBP12 (*gray*) in the presence of 1 equivalent molar Lu^3+^. Inset shows peaks arising from G62. **d** Anchoring point, V2 and T75C (*colored blue*), and G62 (*colored red*) were mapped on the structure of FKBP12

In the crystal structure of FKBP12 (Van Duyne et al. 1991), the well-defined secondary structure starts from V2. We, therefore, omitted G1 and defined the structured region of FKBP12 in all the two-point anchored LBT-attached FKBP12 constructs in this study. The distance between the Cα atoms of the N- and C-terminal residues is around 7 Å in the crystal structure of LBT (Nitz et al. 2004). We searched for a residue about 7 Å in distance from V2 of FKBP12, and found T75. The Cα distance between V2 and T75 was 5.6 Å. Thus we introduced the T75C mutation to FKBP12, and LBT was fused to the N-terminus of the FKBP12 (T75C). A spacer was introduced between the LBT and V2 of FKBP12 to avoid structural distortion and steric hindrance. We prepared constructs containing one- (H-), two- (H-M), three- (H-M-G), four- (H-M-S-G) and five-residue (H-M-G-S-G) linkers, named L1-, L2-, L3-, L4- and L5-FKBP12, respectively (Fig. 1a). These constructs were first screened for their suitability for NMR experiments, based on melting temperature (*T*_m_) measured using differential scanning fluorometry (DSF; Niesen et al. 2007) in the presence of Lu^3+^, since we assumed that *T*_m_ was sensitive to the structural distortion and/or hindrance. Figure 1b shows the unfolding curves, and Table 1 lists the *T*_m_ values of Lu^3+^-bound L1- to L5-FKBP12-rapamycin. The unfolding curves of L3- to L5-FKBP12-rapamycin were almost identical, while those of L1- and L2-FKBP12-rapamycin were shifted to a lower temperature. The melting temperatures of L3- to L5-FKBP12-rapamycin were estimated to be around 72 °C, while those of L1- and L2-FKBP12-rapamycin were lower by 4 and 3.5 °C, respectively. From this observation, L1- and L2-FKBP12-rapamycin were assumed to exhibit structural distortion and/or hindrance. This was also confirmed by the comparison of the ^1^H–^15^N HSQC spectra of these constructs complexed with Lu^3+^ (Fig. 1c). The residues indicating spectral shifts on the attachment of the two-point anchored LBT were located very close to the anchoring points in the case of L3- to L5-FKBP12-rapamycin, while a large shift was observed for G62 (highlighted in Fig. 1d) on the α-helix region close to the N-terminal anchoring point in the case of L1- and L2-FKBP12-rapamycin. From NMR and DSF analyses, a linker with more than three amino acid residues was required for FKBP12 to avoid structural distortion and/or hindrance on the attachment of two-point anchored LBT.

**Table 1:** Melting temperature of L1-, L2-, L3-, L4- and L5-FKBP12 in the presence of Lu^3+^ estimated using DSF

	*T*_m_ (°C)	Δ*T*_m_ (°C)^a^
L1-FKBP12	67.4 (±0.05)	0
L2-FKBP12	68.0 (±0.04)	0.6
L3-FKBP12	71.2 (±0.11)	3.8
L4-FKBP12	71.0 (±0.26)	3.6
L5-FKBP12	70.8 (±0.10)	3.4

^a^Δ*T*_m_: Difference of the *T*_m_ with L1-FKBP12

### NMR analysis and tensor calculation of L3-and L4-FKBP12-rapamycin

**Fig. 2:**
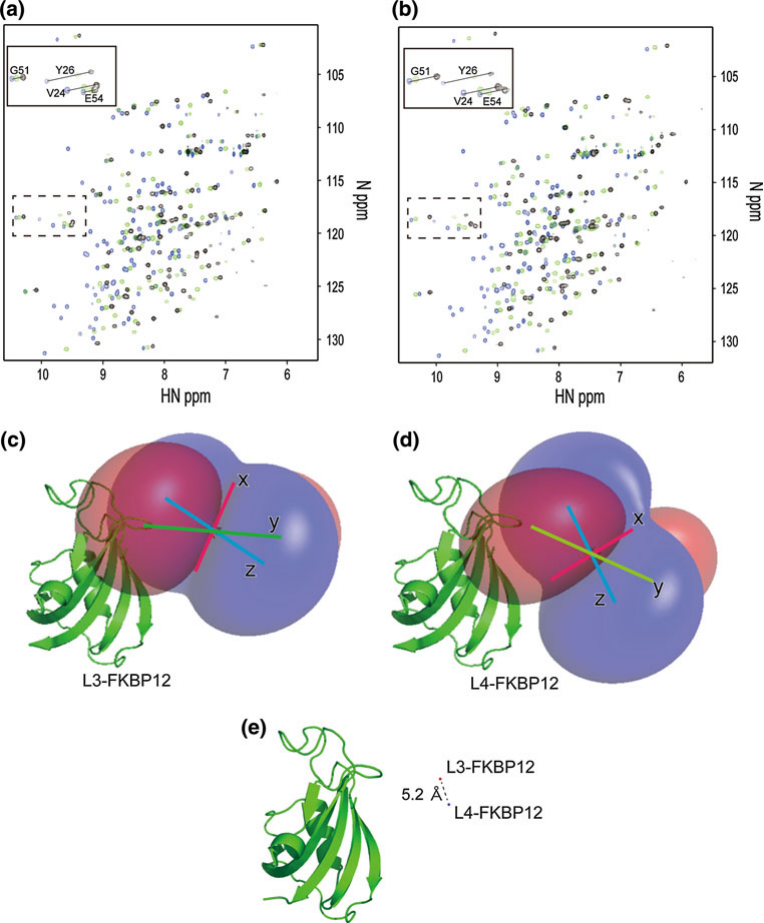
Overlay of the ^1^H–^15^N HSQC spectra of L3-FKBP12 (**a**) and L4-FKBP12 (**b**) in the presence of one equivalent molar Lu^3+^ (*blue*), Dy^3+^ (*green*) and Tb^3+^ (*black*). Graphical views of the PCS isosurface of Dy^3+^ for L3-FKBP12 (**c**) and L4-FKBP12 (**d**). Positive and negative PCS values are indicated by *blue* and *red*, respectively. **e** Metal positions of L3- and L4-FKBP12. Metal positions are shown in ball (*red* for L3 and *blue* for L4)

Considering the results of DSF and NMR analyses, we prepared three two-point anchored LBT-attached constructs, L3- to L5-FKBP12. Using these constructs, we examined the effect of spacer length on the principal axis of the Δχ-tensor and the metal position relative to the target protein. Figure 2a and b show the overlay spectra of Dy^3+^, Lu^3+^ and Tb^3+^-bound L3- (Fig. 2a) and L4-FKBP12-rapamycin (Fig. 2b), respectively. The peak shift pattern of L3-FKBP12-rapamycin was different from that of L4-FKBP12-rapamycin, showing differences in the Δχ-tensor. On the other hand, L4- and L5-FKBP12-rapamycin exhibited similar PCS values (Supplementary Fig. 2), and were assumed to exhibit similar Δχ-tensors and metal positions relative to the target protein. Hence, we estimated the Δχ-tensors for only L3- and L4-FKBP12-rapamycin from the PCS values using the Numbat program (Schmitz et al. 2008). For assignment of the PCS peaks, the ^1^H–^15^N HSQC spectrum of an eleven amino acid (A/F/H/I/K/L/M/R/V/W/Y) inversely labeled sample was also used to reduce spectral complexity (Supplementary Fig. 3). Based on the PCS values from the two lanthanide ions, Tb^3+^ and Dy^3+^, Δχ-tensors for each lanthanide were simultaneously fitted with the common metal position, due to their isomorphous nature, for L3- and L4-FKBP12-rapamycin, respectively (Table 2). The Δχ-tensors were well defined and the correlations between the experimental and back-calculated PCS values were good (Supplemental Fig. 4). This was also supported by the result of Monte-Carlo analysis using the 100 partial PCS data sets in which 30 % of the input data were randomly deleted (Supplemental Fig. 4). Moreover, the magnitudes of the tensors were comparable between L3- and L4-FKBP12-rapamycin as well as to those reported previously (for two-point anchored LBT-attached GB1; Saio et al. 2009, for the p62 PB1 domain; Saio et al. 2010 and for the Grb2 SH2 domain; Saio et al. 2011). Thus, we concluded that the positions of the lanthanide ions as well as the Δχ-tensor parameters for L3- and L4-FKBP12-rapamycin were accurately determined. In contrast to the similarity in magnitude of the Δχ-tensors between L3- and L4-FKBP12-rapamycin, the direction of the principal axes of the Δχ-tensors relative to the attached protein differed by about 30°–40° when compared with the same metal ion (Table 2; Fig. 2c, d). Moreover, the metal positions of these two constructs differed by about 5.2 Å (Fig. 2e). These observations suggest that the PCSs obtained from the two-point anchored LBT-attached proteins with different spacer lengths could be used as independent restraints for structural calculation.

**Table 2:** Δχ-tensor parameters for lanthanide ions in complex with L3-FKBP12-rapamycin, L4-FKBP12-rapamycin, L3-GB1, L4-GB1, L4-Grb2 SH2 and L5-Grb2 SH2 determined on the basis of the crystal structure of the FKBP12-rapamycin complex (PDBID: 1fkb), GB1 (PDBID: 2rpv), Grb2 SH2 domains (PDBID: 1x0n) and the PCS values obtained from L3-FKBP12, L4-FKBP12, L3-GB1, L4-GB1, L4-Grb2 SH2 and L5-Grb2 SH2 signals

	Δχ_ax_^a^	Δχ_rh_^a^	α^b^	*β* ^b^	γ^b^
L3-FKBP12 (Dy^3+^)	23.1 (±1.8)	20.2 (±1.2)	49	106	31
L3-FKBP12 (Tb^3+^)	34.0 (±2.5)	13.4 (±2.1)	53	106	17
L4-FKBP12 (Dy^3+^)	23.1 (±1.6)	19.0 (±0.7)	33	77	38
L4-FKBP12 (Tb^3+^)	29.2 (±1.2)	17.9 (±0.5)	35	69	9
L3-GB1 (Tm^3+^)	−18.5 (±0.9)	−18.0 (±0.4)	66	148	149
L3-GB1 (Tb^3+^)	39.2 (±1.0)	15.9 (±2.1)	97	145	158
L3-GB1 (Er^3+^)	−9.4 (±0.7)	−7.0 (±0.2)	71	138	136
L4-GB1 (Tm^3+^)	−23.6 (±1.2)	−20.0 (±0.7)	85	127	160
L4-GB1 (Tb^3+^)	41.8 (±2.3)	20.4 (±0.6)	94	125	153
L4-GB1 (Er^3+^)	−9.3 (±0.8)	−8.4 (±0.4)	74	115	145
L4-Grb2 SH2 (Dy^3+^)^c^	22.7 (±1.3)	17.6 (±0.7)	106	57	53
L4-Grb2 SH2 (Tb^3+^)^c^	29.2 (±1.7)	16.9 (±0.5)	97	52	34
L4-Grb2 SH2 (Tm^3+^)^c^	−17.5 (±1.6)	−17.1 (±0.5)	99	65	27
L5-Grb2 SH2 (Dy^3+^)	25.1 (±2.2)	21.0 (±1.2)	113	41	51
L5-Grb2 SH2 (Tb^3+^)	30.2 (±2.6)	21.1 (±1.0)	100	42	37
L5-Grb2 SH2 (Tm^3+^)	−19.0 (±1.7)	−19.1 (±1.0)	97	45	34

Δχ-tensor parameters were determined relative to the conformer 1 of the family of NMR structures of FKBP12-rapamycin complex (1fkb.pdb), GB1 (2rpv.pdb) and Grb2 SH2 (1x0n.pdb). Metal ion coordinates were x = 19.155 (±0.340), y = −17.082 (±0.199), z = 13.38 (±0.314) for L3-FKBP12, x = 15.782 (±0.258), y = −19.799 (±0.108), z = 10.386 (±0.263) for L4-FKBP12, x = −3.78 (±0.413), y = −2.12 (±0.548), z = −10.078 (±0.396) for L3-GB1, x = −1.036 (±0.224), y = −3.8 (±0.323), z = −12.954 (±0.219) for L4-GB1, x = −13.093 (±0.186), y = −5.491 (±0.404), z = −5.046 (±0.325) for L4-Grb2 SH2 and x = −13.270 (±0.304), y = −6.009 (±0.409), z = −5.562 (±0.515) for L5-Grb2 SH2. Deviation of the metal position was obtained by the Monte-Carlo protocol using 100 partial PCS data sets in which 30 % of the input data were randomly deleted

^a^Δχ_ax_ and Δχ_rh_ values are in 10^−32^ [m^3^] and error estimates were obtained by the Monte-Carlo protocol using 100 partial PCS data sets in which 30 % of the input data were randomly deleted

^b^Euler angle rotations in ZYZ convention (degrees)

^c^Saio et al. (2011)

### Design, NMR analysis and tensor calculation of L3- and L4-GB1 and L4- and L5-Grb2

To confirm whether the metal position and the principal axes of the Δχ tensor relative to the attached protein could be generally modulated by changing the spacer length between two-point anchored LBT and the attached protein in general, we studied two-point anchored LBT-attached GB1 and Grb2 SH2. In our previous paper, L1-, L2 and L3-GB1 was prepared and the ^1^H–^15^N HSQC spectra were measured in the presence of Tm^3+^, and revealed that L1- and L2-GB1 exhibited double peaks, while single in L3-GB1 (Saio et al. 2009). Hence we assumed that architecture of LBT was disturbed by the steric hindrance due to inappropriate linker length, and we concluded that three residue linker was the minimum spacer length. Moreover, we prepared L3-, L4 and L5-Grb2 SH2 in the previous study (Saio et al. 2011), and showed that L3-Grb2 SH2 exhibited broad NMR signals, while sharp signals in L4- and L5-Grb2 SH2 domain. From these observations, we assumed that the minimum spacer length of Grb2 SH2 was four residues. Based both on the previous result for GB1 and Grb2 SH2, and the present result of FKBP12, we prepared L5-Grb2 SH2 and L4-GB1 constructs for the Δχ-tensor analysis. The principal axes of the Δχ-tensors and the metal positions for L3- and L4-GB1, and L4- and L5-Grb2 SH2 were evaluated in a manner similar to that for FKBP12-rapamycin. The magnitudes of the Δχ-tensors were comparable among the L3- and L4-GB1, L4- and L5-Grb2 SH2, and FKBP12-rapamycin as well as to those reported previously (for two-point anchored LBT-attached GB1; Saio et al. 2009, for the p62 PB1 domain; Saio et al. 2010 and for the Grb2 SH2 domain; Saio et al. 2011). It could be concluded that the position of the lanthanide ion as well as the Δχ-tensor parameters for L3- and L4-GB1, and L4- and L5-Grb2 SH2 were accurately determined, since the Δχ-tensor parameters were well defined and the correlations between the experimental and back-calculated PCS values were good (Supplemental Fig. 5 and Supplemental Fig. 6). This was also supported by the result of Monte-Carlo analysis using the 100 partial PCS data sets in which 30 % of the input data were randomly deleted (Supplemental Fig. 5 and Supplemental Fig. 6). The principal axes of the Δχ-tensors differed by about 20°–30° between L3- and L4-GB1, and by about 10°–20° between L4- and L5-Grb2 SH2 for the same metal ions (Table 2). Moreover, the metal positions in L3- and L4-GB1 differed by about 4.3 Å (Supplemental Fig. 6), while those in the L4- and L5-Grb2 SH2 domains differed by about 0.7 Å (Supplemental Fig. 5). Thus, both the principal axes of the Δχ-tensor and the metal positions relative to the attached protein can be modulated by changing the spacer length between the two-point anchored LBT and the attached protein.

### PCS-restraint based rigid body docking of the FKBP12-rapamycin-FRB complex

**Fig. 3:**
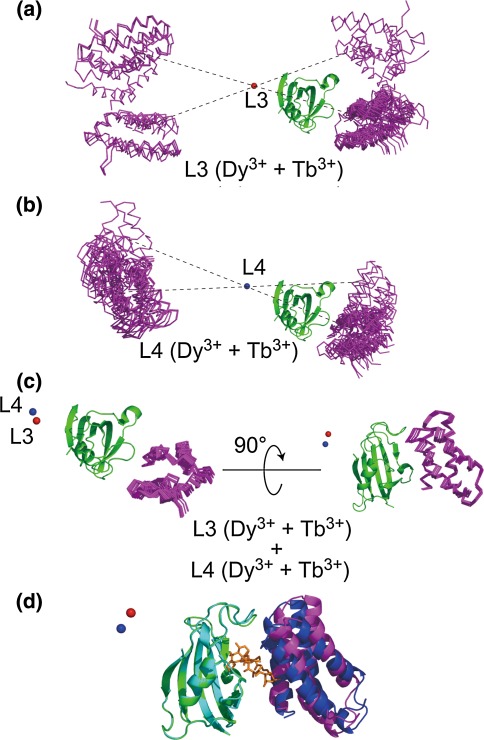
The PCS-based docking structure between the FKBP12-rapamycin and FRB domains. Since PCS data were not obtained for rapamycin, rapamycin was omitted during the structure calculation. **a** Calculated FKBP12-FRB complex structure based on PCS data from L3-FKBP12 using two metals, both Dy^3+^ and Tb^3+^. **b** Calculated FKBP12-FRB complex structure based on PCS data from L4-FKBP12 using two metals, both Dy^3+^ and Tb^3+^. **c** Calculated FKBP12-FRB complex structure based on PCS data from both L3- and L4-FKBP12 using two metals, both Dy^3+^ and Tb^3+^. Through (**a**) to (**c**), obtained structures were superimposed on FKBP12 moiety. In (**a**), (**b**) and (**c**), metal positions are shown in ball (*red* for L3 and *blue* for L4), FKBP12 in *green* ribbon and FRB in magenta stick. **d** *Ribbon* representation of the PCS-based structure (*green* for FKBP12 and magenta for FRB) and the crystal structure of FKBP12/rapamycin/FRB ternary complex (Liang et al. 1999, 1fap.pdb; cyan ribbon for FKBP12, *orange* stick for rapamycin and blue ribbon for FRB). The lowest energy structure of the PCS-based structure of the FKBP12-FRB complex was superimposed on FKBP12 moiety of the crystal structure of the ternary complex (Liang et al. 1999, 1fap.pdb). The main chain atom RMSD of the FKBP12 moiety in the binary and the ternary complexes was estimated to be 0.5 Å. The main chain atom RMSD of the FRB moiety in the ternary complex of the crystal structure and the PCS-based NMR structure was estimated to be 2.9 Å

We next studied whether the present approach could be used for resolving the degeneracy problem in PCS-based structure calculation. We initially confirmed that FRB moiety of FRB/rapamycin/L3-FKBP12 ternary complex exhibited different PCS pattern as compared to the FRB/rapamycin/L4-FKBP12 (Supplementary Fig. 7). Next, the structure of the FKBP12-FRB complex was calculated based solely on PCS restraints, and compared with the crystal structure (Liang et al. 1999). First, rigid body docking calculations were performed for L3- and L4-FKBP12 separately, using two PCS data sets derived from Dy^3+^ and Tb^3+^. The docking structure determined using PCS data sets derived from two lanthanide ions still affords four degenerate solutions (Fig. 3a, b). This is consistent with our previous result (Saio et al. 2010).

PCSs can also be expressed by Eq. (2) (Bertini et al. 2002):

2 $$ \Updelta \delta^{\text{pcs}} = \frac{1}{{12\pi {\text{r}}^{3} }}\left[ {\Updelta \chi_{{{\text{ax}} }} \frac{{2{\text{z}}^{2} - {\text{x}}^{2} - {\text{y}}^{2} }}{{{\text{r}}^{2} }} + \frac{3}{2}\Updelta \chi_{{{\text{rh}} }} \frac{{{\text{x}}^{2} - {\text{y}}^{2} }}{{{\text{r}}^{2} }}} \right] $$

where x, y and z are the Cartesian coordinates of the nuclear spin in the Δχ-tensor frame, r is the distance between the nuclear spin and the paramagnetic centre, and Δχ_ax_ and Δχ_rh_ are the axial and rhombic components of the Δχ-tensor. From Eq. (2), PCSs from a single metal exhibit eight degenerated solutions, in principle, since PCS values do not depend on the sign of the x, y, and z axes. The degenerate solutions can be eliminated in case of calculations based on PCS data sets from multiple lanthanide ions, since the x, y and z directions of the principal axes of the Δχ-tensor vary among the lanthanide ions fixed in the same position (Saio et al. 2009, 2010, 2011). In this study, the directions of the x and y axes mainly differed by 20–30° between Tb^3+^ and Dy^3+^, which was represented as the difference in the γ term of the Euler angles (Table 2), thus restricting four solutions (Fig. 3a, b).

We next combined the PCS restraints derived from the L3- and L4-FKBP systems, and performed a rigid body docking calculation based on four PCS data sets: the PCSs of Dy^3+^ and Tb^3+^ observed for both the L3- and L4-FKBP12 system. The Δχ-tensor parameters were well defined and the correlations between the experimental and back-calculated PCS values were good (Supplemental Fig. 8). Figure 3c shows an overlay of the 20 lowest energy structures of the FKBP12-rapamycin-FRB complex superimposed on FKBP12 region. FRB regions were converged with the main chain atom RMSD of 0.69 Å (±0.58 Å), and position of the FRB relative to FKBP12 were well defined. Moreover, combined use of the PCS data sets with different lanthanide ion positions as well as the different directions of the tensor axes successfully resolved the degeneracy, thus structural determination of the FKBP12-FRB complex can be achieved. We also show an overlay of the lowest energy structure of the PCS-based FKBP12-FRB complex superimposed on FKBP12 region of the ternary complex determined by X-ray crystallography (Liang et al. 1999, 1fap.pdb) in Fig. 3d. The position and orientation of FRB domain relative to FKBP12 correspond well to those of the structure determined by X-ray crystallography (Liang et al. 1999, 1fap.pdb). Since we omitted the rapamycin during the docking calculation, relative orientation between FKBP12 and FRB could be determined solely by PCS-restraints not by complementary of the surface shape. FRB region of PCS-based structures were converged with the main chain atom RMSD of 2.9 Å, which validates the PCS-based structure obtained using the present method. Slight difference between the complex structures obtained by PCS and crystal might be caused by the limitation of the PCS-based docking, presumably structural change of FKBP12 according to association with FRB (the main chain atom RMSD of the FKBP12 moiety in the binary and the ternary complexes was estimated to be 0.5 Å) and/or the experimental error of the PCS values etc. It should be also noted that the correlations between the experimental and back-calculated PCS values were good (Supplemental Fig. 8), which supports the compatibility of the docking structure.

## Discussion

In the present study, we demonstrated that two-point anchored LBT accommodates at least two sets of different spacer lengths, which change the orientation of the LBT, thus changing the direction of the principal axis of the Δχ-tensor and the metal position relative to the attached protein. We applied this method to three proteins, FKBP12, GB1 and Grb2 SH2, and showed that the two-point anchored LBT-attached target proteins with different spacer length with minimum and ‘minimum plus one’ spacer length could change the orientation of the principal axes of the Δχ-tensor and the metal position, relative to the attached proteins. Moreover, we demonstrated that the degeneracy problem could be overcome by the use of the PCS data sets derived from the constructs with different spacer lengths.

In our previous study, we reported a vector to construct an expression plasmid for a two-point anchored LBT-attached protein. The vector codes the N-terminus GST for affinity purification, the TEV protease cleavage site for GST-tag removal, the LBT and multiple cloning sites for the introduction of the cDNA fragment of the target protein (Saio et al. 2010 and Supplementary Fig. 1). By changing the primer used for amplification of the cDNA fragment, the spacer length between the two-point anchored LBT and the target protein can be easily changed. However, it is to be noted that a longer spacer would result in increased mobility of the lanthanide ion relative to the protein framework and reduction of the anisotropic paramagnetic effect. From the present results for FKBP12 and GB1, the appreciable changes in the direction of the principal axis of the Δχ-tensor and the metal position were produced between the three (minimum) and four (minimum plus one) amino acid residue linkers. In case of FKBP12, both constructs with the four (minimum plus one) and five (minimum plus two) residue linker exhibited almost identical PCS values and are assumed to possess the almost identical principal axis of the Δχ-tensor and the metal position.

Therefore, a minimum spacer length should be determined. Table 3 lists Cα atom distances between the N-terminus residue of the target and the anchoring point disulfide bond, and the minimum spacer length between the target and two-point anchored LBT applied to date. It could be empirically assumed that the minimal spacer length was three residues if the Cα atom distance was around 6 Å. For the longer Cα atom distances, a longer spacer would be required. Further analysis is required to obtain information about the correlation between the Cα atom distance and the spacer length where the Cα atom distance is much longer than 6 Å. At least, this empirical ‘6 Å-three residue linker’ rule for the two-point anchored LBT will be helpful for the design of the anchoring point to be mutated to Cys. Once this anchoring point is designed, the stability of the constructs with the minimum and the minimum plus one spacer lengths can be determined by DSF and/or NMR.

**Table 3:** Spacer length between the two-point anchored LBT and target proteins, and the distance between the Cα atoms of N-terminus residue of the target and the anchoring residue disulfide bond

	Anchoring point	Cα atom distance (Å)	Minimal spacer length
GB1	M1-E19C	6.1	3^a^
p62 PB1 domain	S3-C26	6.0	3^b^
FKBP12	V2-T75C	5.6	3^c^
Grb2 SH2 domain	W60-M73C	9.9	4^d^

^a^Saio et al. (2009)

^b^Saio et al. (2010)

^c^Present study

^d^Saio et al. (2011)

## Electronic supplementary material

Below is the link to the electronic supplementary material.

Supplementary material 1 (DOCX 43 kb)

Supplementary material 2 (DOCX 17 kb)

Supplementary material 3 (TIFF 667 kb)

Supplementary material 4 (TIFF 1428 kb)

Supplementary material 5 (TIFF 1248 kb)

Supplementary material 6 (TIFF 1677 kb)

Supplementary material 7 (TIFF 2180 kb)

Supplementary material 8 (TIFF 1521 kb)

Supplementary material 9 (TIFF 1533 kb)

Supplementary material 10 (TIFF 892 kb)

Supplementary material 11 (TIFF 1884 kb)

Supplementary material 12 (TIFF 1213 kb)

Supplementary material 13 (DOCX 29 kb)

## Abbreviations

NMR: Nuclear Magnetic Resonance
PCS: Pseudo contact shift
RDC: Residual dipolar coupling
FKBP12: FK-506 binding protein 12
FRB: FKBP12 rapamycin binding
DSF: Differential scanning fluorometry
LBT: Lanthanide-binding peptide tag
