# Supplementary material for: Convenient method for resolving degeneracies due to symmetry of the magnetic susceptibility tensor and its application to pseudo contact shift-based protein–protein complex structure determination
Source: J Biomol NMR. 2012 Apr 10;53(1):53–63. doi: 10.1007/s10858-012-9623-8 (PMC3351616; doi:10.1007/s10858-012-9623-8)
Supplement: Supplementary file 1 — Supplementary material 1 (DOCX 43 kb) [file 10858_2012_9623_MOESM1_ESM.docx]

*Supplementary Table 1: PCS values of amide protons observed for L3- and L4-FKBP12-rapamycin in complex with Dy^3+^and Tb^3+^.*

| Residue number | L3 (Dy^3+^)  (ppm) | L3 (Tb^3+^)  (ppm) | L4 (Dy^3+^)  (ppm) | L4 (Tb^3+^)  (ppm) |
| --- | --- | --- | --- | --- |
| 3 | -0.964 | -2.135 | -1.05 | -2.786 |
| 4 | -0.367 |  | -0.679 |  |
| 5 | -0.668 | -2.011 | -1.008 | -2.743 |
| 7 | -0.228 | -0.946 | -0.3 | -1.055 |
| 8 | -0.154 | -0.697 | -0.153 | -0.687 |
| 21 | -0.058 | -0.138 | -0.058 | -0.205 |
| 22 | -0.106 | -0.219 | -0.109 | -0.296 |
| 23 | -0.112 | -0.254 | -0.122 | -0.317 |
| 24 | -0.186 | -0.321 | -0.186 | -0.372 |
| 25 | -0.238 | -0.44 | -0.241 | -0.487 |
| 26 | -0.340 | -0.478 | -0.334 | -0.518 |
| 27 | -0.551 | -0.775 | -0.567 | -0.828 |
| 28 | -0.652 | -0.751 | -0.598 | -0.741 |
| 29 |  |  | -0.978 | -1.156 |
| 46 | -0.213 | -0.324 | -0.212 | -0.37 |
| 47 | -0.167 | -0.242 | -0.178 | -0.314 |
| 48 | -0.141 | -0.236 | -0.141 | -0.315 |
| 49 | -0.103 | -0.163 | -0.105 | -0.264 |
| 56 | -0.272 | -0.302 | -0.227 | -0.451 |
| 57 | -0.169 | -0.154 | -0.155 | -0.435 |
| 58 | -0.299 | -0.363 | -0.251 | -0.675 |
| 59 | -0.299 | -0.444 | -0.288 | -0.702 |
| 60 | -0.184 | -0.326 | -0.185 | -0.573 |
| 61 | -0.103 | -0.303 | -0.149 | -0.645 |
| 62 | -0.198 | -0.532 | -0.238 | -0.883 |
| 63 | -0.213 |  | -0.235 | -0.778 |
| 64 | -0.102 | -0.376 | -0.119 | -0.617 |
| 65 | -0.078 | -0.455 | -0.084 | -0.699 |
| 71 | -0.144 | -0.577 | -0.131 | -0.591 |
| 72 | -0.348 | -0.859 | -0.424 | -0.996 |
| 73 | -0.602 |  | -0.863 |  |
| 74 | -0.922 | -1.598 | -1.032 |  |
| 76 | -1.71 | -2.102 | -1.25 | -2.001 |
| 79 | -0.294 | 0.276 | -0.078 | -0.279 |
| 80 | -0.351 | -0.103 | -0.183 | -0.531 |
| 81 | -0.416 | -0.196 | -0.22 | -0.473 |
| 97 |  |  |  | -1.369 |
| 99 | -1.174 | -1.647 | -1.134 | -1.745 |
| 100 | -0.76 | -1.084 | -0.813 | -1.167 |
| 101 | -0.512 | -0.953 | -0.594 | -1.129 |
| 102 | -0.307 | -0.579 | -0.33 | -0.644 |
| 103 | -0.186 | -0.482 | -0.185 | -0.533 |
| 104 | -0.151 | -0.334 | -0.147 | -0.375 |
| 105 | -0.139 | -0.269 | -0.108 | -0.3 |
| 106 | -0.067 | -0.185 | -0.055 | -0.218 |
| 107 | -0.067 | -0.161 | -0.06 | -0.211 |

*Supplementary Table 2: PCS values of amide proton observed for the FRB domain of L3- and L4-FKBP12-rapamycin-FRB in complex with Dy^3+^and Tb^3+^.*

| Residue number | L3 (Dy^3+^)  (ppm) | L3 (Tb^3+^)  (ppm) | L4 (Dy^3+^)  (ppm) | L4 (Tb^3+^)  (ppm) |
| --- | --- | --- | --- | --- |
| 2017 | -0.118 | -0.14 | -0.08 | -0.157 |
| 2019 | -0.028 | 0 | -0.026 | -0.016 |
| 2020 | -0.083 | -0.003 | -0.04 | -0.021 |
| 2021 | -0.107 | -0.053 | -0.074 | -0.082 |
| 2023 |  | -0.115 | -0.082 | -0.12 |
| 2024 | -0.171 |  | -0.122 | -0.188 |
| 2040 | -0.062 | -0.054 |  |  |
| 2041 | -0.06 | -0.017 | -0.057 | -0.09 |
| 2042 | -0.082 | -0.031 | -0.047 | -0.085 |
| 2043 | -0.07 | -0.026 | -0.032 | -0.067 |
| 2044 | -0.078 | -0.037 | -0.037 | -0.057 |
| 2046 | -0.005 | 0.015 | 0.005 | -0.011 |
| 2048 | -0.032 | -0.023 | -0.006 | -0.06 |
| 2049 |  |  | -0.07 | -0.066 |
| 2050 |  | -0.007 | 0.022 | -0.027 |
| 2051 | -0.016 | -0.063 | -0.003 |  |
| 2054 | -0.059 | -0.059 | -0.048 | -0.077 |
| 2055 | -0.067 | -0.073 | -0.055 | -0.089 |
| 2056 | -0.042 | -0.035 | -0.047 | -0.067 |
| 2057 | -0.062 | -0.05 | -0.044 | -0.064 |
| 2058 | -0.072 | -0.064 | -0.058 | -0.081 |
| 2059 | -0.055 | -0.052 | -0.043 | -0.066 |
| 2060 | -0.054 | -0.047 | -0.041 | -0.056 |
| 2061 | -0.062 | -0.051 | -0.049 | -0.064 |
| 2063 | -0.07 | -0.051 | -0.045 | -0.062 |
| 2064 | -0.057 | -0.047 | -0.045 | -0.066 |
| 2067 | -0.078 | -0.061 | -0.055 | -0.072 |
| 2068 | -0.074 | -0.067 | -0.055 | -0.078 |
| 2070 | -0.097 | -0.094 | -0.078 | -0.11 |
| 2071 | -0.1 | -0.095 | -0.064 | -0.098 |
| 2072 | -0.088 | -0.091 | -0.07 | -0.095 |
| 2073 | -0.089 | -0.097 | -0.072 | -0.106 |
| 2074 | -0.104 | -0.114 | -0.079 | -0.116 |
| 2075 | -0.096 | -0.1 | -0.079 | -0.114 |
| 2077 | -0.097 | -0.113 | -0.084 | -0.121 |
| 2078 |  |  | -0.08 | -0.112 |
| 2079 | -0.086 | -0.094 | -0.075 | -0.111 |
| 2081 |  |  |  | -0.103 |
| 2084 | -0.087 | 0.02 | -0.019 | -0.015 |
| 2085 | -0.064 | -0.076 |  |  |
| 2086 | -0.056 | -0.074 | -0.067 | -0.072 |
| 2087 |  | -0.081 | -0.057 | -0.065 |
| 2088 |  |  | 0.003 | -0.008 |
| 2089 | -0.098 | -0.077 | -0.065 | -0.072 |
| 2090 | -0.103 | -0.077 | -0.065 | -0.066 |
| 2091 | -0.124 | -0.088 | -0.082 | -0.071 |
| 2092 | -0.143 | -0.093 | -0.082 | -0.074 |
| 2093 | -0.17 | -0.125 | -0.114 | -0.102 |
| 2094 | -0.244 | -0.172 | -0.155 | -0.135 |
| 2096 | -0.206 | -0.166 | -0.15 | -0.151 |
| 2097 | -0.183 | -0.163 | -0.136 | -0.16 |
| 2098 | -0.202 | -0.189 | -0.154 | -0.192 |
| 2100 | -0.143 | -0.144 | -0.112 | -0.15 |
| 2101 | -0.126 | -0.147 | -0.103 | -0.159 |
| 2103 | -0.119 | -0.139 | -0.11 | -0.161 |
| 2104 | -0.091 | -0.136 | -0.075 | -0.151 |
| 2105 | -0.075 | -0.135 | -0.067 | -0.163 |
| 2106 | -0.098 | -0.145 | -0.086 | -0.174 |
| 2107 | -0.096 | -0.131 | -0.093 | -0.153 |
| 2108 | -0.079 | -0.14 | -0.076 |  |
| 2109 | -0.077 | -0.124 | -0.08 | -0.15 |
| 2110 | -0.101 | -0.132 | -0.085 | -0.161 |
| 2111 | -0.167 | -0.158 | -0.139 | -0.19 |
| 2112 |  |  | -0.128 | -0.192 |
| 2113 |  | -0.123 | -0.111 | -0.144 |

*Supplementary Table 3: PCS values of amide proton observed for L5-Grb2 SH2 domain in complex with Dy^3+^,* *Tb^3+^and Tm^3+^.*

| Residue number | Dy^3+^ (ppm) | Tb^3+^ (ppm) | Tm^3+^ (ppm) |
| --- | --- | --- | --- |
| 68 | 0.05 | -1.04 | 1.15 |
| 71 |  |  | 1.81 |
| 83 | -0.6 | -1.18 | 0.94 |
| 84 | -0.49 | -1.39 | 1.03 |
| 94 | 0.29 | -0.09 | 0.05 |
| 95 | 0.18 | -0.27 | 0.23 |
| 96 | 0.11 | -0.66 | 0.54 |
| 97 | -0.04 | -0.62 | 0.55 |
| 98 |  |  | 0.93 |
| 99 | -0.38 | -0.96 | 0.79 |
| 100 | -0.58 | -1.08 | 0.86 |
| 101 | -0.46 |  |  |
| 104 | -0.44 |  |  |
| 105 | -0.44 | -0.82 | 0.69 |
| 106 | -0.38 | -0.82 | 0.69 |
| 107 | -0.18 | -0.6 | 0.53 |
| 108 | -0.12 | -0.63 | 0.54 |
| 109 | -0.01 | -0.35 | 0.32 |
| 110 | 0.06 | -0.37 | 0.3 |
| 111 | 0.04 | -0.23 | 0.19 |
| 112 | 0.07 | -0.13 |  |
| 113 |  | -0.1 | 0.04 |
| 117 | 0.06 | -0.06 | 0.03 |
| 118 | 0.06 | -0.11 | 0.09 |
| 119 | 0.04 | -0.19 | 0.14 |
| 122 | -0.01 | -0.16 | 0.14 |
| 123 | 0 | -0.14 | 0.13 |
| 124 | 0.01 | -0.11 | 0.08 |
| 125 | 0.03 | -0.13 | 0.09 |
| 126 | 0.05 | -0.08 | 0.05 |
| 127 | 0.07 | -0.1 | 0.06 |
| 130 | 0.07 | -0.14 | 0.09 |
| 131 | 0.05 | -0.2 | 0.14 |
| 133 | 0 | -0.24 | 0.17 |
| 134 | -0.02 | -0.22 | 0.15 |
| 136 | -0.07 |  | 0.21 |
| 137 | -0.06 | -0.23 | 0.19 |
| 138 | -0.08 | -0.25 | 0.18 |
| 141 | -0.11 | -0.32 | 0.26 |
| 146 | -0.15 | -0.33 | 0.27 |
| 147 | -0.21 | -0.41 | 0.33 |
| 148 |  |  | 0.33 |
| 149 | -0.34 | -0.67 | 0.54 |
| 150 | -0.32 | -0.58 | 0.43 |

*Supplementary Table 4: PCS values of amide proton observed for L4-GB1 in complex with Er^3+^,* *Tb^3+^and Tm^3+^.*

| Residue number | Er^3+^ (ppm) | Tb^3+^ (ppm) | Tm^3+^ (ppm) |
| --- | --- | --- | --- |
| 22 | 0.816 |  | 2.38 |
| 23 | 0.44 |  | 1.266 |
| 24 | 0.362 |  |  |
| 25 | 0.201 | -0.736 | 0.659 |
| 26 | 0.159 | -0.545 | 0.529 |
| 27 | 0.099 | -0.362 | 0.357 |
| 28 | 0.077 | -0.266 | 0.285 |
| 29 | 0.035 | -0.154 | 0.182 |
| 30 | 0.033 | -0.125 | 0.164 |
| 31 | 0.045 | -0.156 | 0.202 |
| 32 | 0.073 | -0.225 | 0.264 |
| 33 | 0.123 | -0.408 | 0.412 |
| 34 | 0.202 | -0.61 | 0.622 |
| 35 | 0.301 | -1.032 | 0.958 |
| 36 | 0.548 | -1.821 | 1.724 |
| 39 | 1.053 |  | 3.351 |
| 40 |  |  | 2.289 |
| 41 | 0.272 |  | 1.269 |
| 42 | 0.178 |  | 0.689 |
| 43 | 0.095 | -0.766 | 0.56 |
| 44 | 0.098 | -0.974 | 0.722 |
| 45 | 0.22 |  | 1.158 |
| 46 | 0.178 | -0.949 | 0.822 |
| 47 | 0.058 | -0.748 | 0.612 |
| 48 | 0.068 | -0.931 | 0.807 |
| 49 | 0.138 | -0.918 | 0.848 |
| 50 | 0.071 | -0.556 | 0.539 |
| 51 | -0.002 | -0.379 | 0.405 |
| 52 | 0.039 | -0.409 | 0.51 |
| 53 | 0.05 | -0.378 | 0.431 |
| 54 | 0 | -0.201 | 0.261 |
| 55 | -0.02 | -0.069 | 0.206 |
| 56 | 0.006 | -0.096 | 0.22 |
| 57 | 0.005 | -0.045 | 0.163 |
| 58 | 0.024 | -0.14 | 0.212 |
| 59 | 0.039 | -0.148 | 0.158 |
| 60 | 0.044 | -0.202 | 0.234 |
| 61 | 0.024 | -0.226 | 0.258 |
| 62 | 0.058 | -0.272 | 0.254 |
| 63 | 0.098 | -0.423 | 0.369 |
| 64 | 0.105 | -0.428 | 0.36 |
| 65 | 0.172 | -0.643 | 0.534 |
| 66 | 0.147 | -0.514 | 0.422 |
| 67 | 0.166 | -0.535 | 0.438 |
| 68 | 0.218 | -0.694 | 0.562 |
| 69 | 0.266 | -0.868 | 0.707 |
| 70 | 0.259 | -0.874 | 0.691 |
| 71 | 0.261 | -0.934 | 0.815 |
| 72 | 0.14 | -0.556 | 0.483 |
| 73 | 0.123 | -0.467 | 0.439 |
| 74 | 0.075 | -0.319 | 0.292 |
| 75 | 0.057 | -0.23 | 0.231 |

*Supplementary Table 5: PCS values of amide proton observed for L3-GB1 in complex with Er^3+^,* *Tb^3+^and Tm^3+^.*

| Residue number | Er^3+^ (ppm) | Tb^3+^ (ppm) | Tm^3+^ (ppm) |
| --- | --- | --- | --- |
| 22 | 0.67 |  | 1.93 |
| 23 | 0.37 |  |  |
| 25 | 0.19 |  |  |
| 26 | 0.16 | -0.57 | 0.49 |
| 27 | 0.1 | -0.38 | 0.33 |
| 28 | 0.09 | -0.33 | 0.28 |
| 29 | 0.05 | -0.21 | 0.19 |
| 30 | 0.05 | -0.2 | 0.2 |
| 31 | 0.07 | -0.25 | 0.23 |
| 32 | 0.1 | -0.33 | 0.3 |
| 33 | 0.13 | -0.47 | 0.4 |
| 34 | 0.22 | -0.7 | 0.61 |
| 35 | 0.29 | -1.02 | 0.86 |
| 36 | 0.5 | -1.71 | 1.57 |
| 40 | 0.24 |  |  |
| 42 | 0.09 |  | 0.44 |
| 43 |  | -0.51 | 0.35 |
| 44 | 0.07 |  | 0.51 |
| 45 |  |  | 0.86 |
| 46 | 0.14 | -0.79 | 0.63 |
| 47 | 0.06 | -0.68 | 0.5 |
| 48 | 0.1 |  | 0.71 |
| 49 | 0.16 |  |  |
| 50 | 0.09 | -0.59 | 0.49 |
| 51 | 0.05 | -0.5 | 0.43 |
| 52 | 0.1 | -0.57 | 0.53 |
| 53 | 0.09 | -0.49 | 0.45 |
| 54 | 0.05 | -0.34 | 0.31 |
| 55 | 0.04 | -0.28 |  |
| 56 | 0.05 | -0.3 | 0.3 |
| 57 | 0.04 | -0.21 | 0.23 |
| 58 | 0.05 | -0.26 | 0.25 |
| 59 | 0.04 | -0.22 | 0.19 |
| 60 | 0.05 | -0.24 | 0.21 |
| 61 |  | -0.23 | 0.19 |
| 62 | 0.05 | -0.25 | 0.2 |
| 63 | 0.08 | -0.37 | 0.29 |
| 64 | 0.08 | -0.32 | 0.25 |
| 65 |  | -0.49 | 0.37 |
| 66 | 0.09 | -0.3 | 0.24 |
| 67 | 0.1 | -0.29 | 0.23 |
| 68 | 0.15 | -0.45 | 0.34 |
| 69 | 0.19 |  | 0.46 |
| 70 | 0.2 | -0.66 | 0.53 |
| 71 | 0.22 | -0.8 | 0.65 |
| 72 | 0.12 | -0.48 | 0.38 |
| 73 | 0.12 | -0.46 | 0.38 |
| 74 | 0.07 | -0.3 | 0.25 |
| 75 | 0.06 | -0.26 | 0.22 |
