# Supplementary material for: Convenient method for resolving degeneracies due to symmetry of the magnetic susceptibility tensor and its application to pseudo contact shift-based protein–protein complex structure determination
Source: J Biomol NMR. 2012 Apr 10;53(1):53–63. doi: 10.1007/s10858-012-9623-8 (PMC3351616; doi:10.1007/s10858-012-9623-8)
Supplement: Supplementary file 2 — Supplementary material 2 (DOCX 17 kb) [file 10858_2012_9623_MOESM2_ESM.docx]

**Supplementary figure legends**

Supplementary Fig.1: Plasmid map of pGTL.

Supplementary Fig. 2: Overlay of the ^1^H-^15^N HSQC spectra of L4-FKBP12-rapamycin and L5-FKBP12-rapamycin in the presence of 1 equivalent molar Lu^3+^ (green for L4- and black for L5-FKBP12-rapamycin) and Tb^3+^ (blue for L4- and red for L5-FKBP12-rapamycin).

Supplementary Fig. 3: Overlay of the ^1^H-^15^N HSQC spectra of eleven amino acids (A/F/H/I/K/L/M/R/V/W/Y) inversely labeled L3-FKBP12-rapamycin (upper panel) and L4-FKBP12-rapamycin (lower panel) in the presence of 1 equivalent molar Lu^3+^ (blue), Dy^3+^ (green) and Tb^3+^ (black).

Supplementary Fig. 4: (a) Comparison between the experimental and the back-calculated PCS of backbone amide protons observed for L3- and L4-FKBP12-rapamycin, in the presence of Dy^3+^ and Tb^3+^. The ideal correlations are indicated in lines. The theoretical PCS values were calculated based on the Δχ-tensor parameters for the L3- and L4-FKBP12-rapamycin (Table 2). (b) Orientation of the principal axis of the magnetic susceptibility tensor of Dy^3+^ and Tb^3+^, determined based on the PCS values from L3- and L4-FKBP12-rapamycin. The orientations are visualized in Sanson-Flamsteed projection (Bugayevskiy and Snyder, 1995). The plots show the points where the principal axes of the Δχ-tensor penetrate the sphere. The convention |z| > |y| > |x| was used to name the axes, which occasionally cause the swapping between the z- and y-axes of the tensors when their magnitudes were similar. 100 sets of plots represent the result of Monte-Carlo analysis using the 100 partial PCS data sets in which 30 % of the input data were randomly deleted.

Supplementary Fig. 5: (a) Comparison between the experimental and the back-calculated PCS of backbone amide protons observed for the L5-Grb2 SH2, in the presence of Dy^3+^, Tb^3+^ and Tm^3+^. The ideal correlations are indicated in lines. The theoretical PCS values were calculated based on the Δχ-tensor parameters for the L5-Grb2 SH2 (Table 2). (b) Orientation of the principal axis of the magnetic susceptibility tensor of Dy^3+^, Tb^3+^ and Tm^3+^, determined based on the PCS values from the L4-Grb2 SH2 (upper panel) (Saio et al., 2011) and L5-Grb2 SH2 (lower panel). The orientations are visualized in Sanson-Flamsteed projection (Bugayevskiy and Snyder, 1995). The plots show the points where the principal axes of the Δχ-tensor penetrate the sphere. The convention |z| > |y| > |x| was used to name the axes, which occasionally cause the swapping between the z- and y-axes of the tensors when their magnitudes were similar. 100 sets of plots represent the result of Monte-Carlo analysis using the 100 partial PCS data sets in which 30 % of the input data were randomly deleted. (c) Metal positions of L4- (blue sphere) and L5-Grb2 SH2 domain (red sphere). Metal positions are shown in small spheres.

Supplementary Fig. 6: (a) Comparison between the experimental and the back-calculated PCS of backbone amide protons observed for L4-GB1, in the presence of Er^3+^, Tb^3+^ and Tm^3+^. The ideal correlations are indicated in solid lines. The theoretical PCS values were calculated based on the Δχ-tensor parameters for the L4-GB1 (Table 2). (b) Orientation of the principal axis of the magnetic susceptibility tensor of Er^3+^, Tb^3+^ and Tm^3+^, determined based on the PCS values from L4-GB1. The orientations are visualized in Sanson-Flamsteed projection (Bugayevskiy and Snyder, 1995). The plots show the points where the principal axes of the Δχ-tensor penetrate the sphere. The convention |z| > |y| > |x| was used to name the axes, which occasionally cause the swapping between the z- and y-axes of the tensors when their magnitudes were similar. 100 sets of plots represent the result of Monte-Carlo analysis using the 100 partial PCS data sets in which 30 % of the input data were randomly deleted. (c) Comparison between the experimental and the back-calculated PCS of backbone amide protons observed for L4-GB1, in the presence of Er^3+^, Tb^3+^ and Tm^3+^. The ideal correlations are indicated in solid lines. The theoretical PCS values were calculated based on the Δχ-tensor parameters for the L4-GB1 (Table 2). (d) Orientation of the principal axis of the magnetic susceptibility tensor of Er^3+^, Tb^3+^ and Tm^3+^, determined based on the PCS values from L4-GB1. The orientations are visualized in Sanson-Flamsteed projection (Bugayevskiy and Snyder, 1995). The plots show the points where the principal axes of the Δχ-tensor penetrate the sphere. The convention |z| > |y| > |x| was used to name the axes, which occasionally cause the swapping between the z- and y-axes of the tensors when their magnitudes were similar. 100 sets of plots represent the result of Monte-Carlo analysis using the 100 partial PCS data sets in which 30 % of the input data were randomly deleted.(e) Metal positions of L3- and L4-GB1. Metal positions are shown in ball (red for L3 and blue for L4).

Supplementary Fig. 7: Overlay of the ^1^H-^15^N HSQC spectra of ^15^N FRB/rapamycin/non-label L3-FKBP12 ternary complex (upper panel) and ^15^N FRB/rapamycin/non-label L3-FKBP12 ternary complex (lower panel) in the presence of 1 equivalent molar Lu^3+^ (blue), Dy^3+^ (green) and Tb^3+^ (black).

Supplementary Fig. 8: Comparison between experimental and back-calculated PCS of backbone amide protons observed for L3- and L4-FKBP12-rapamycin-FRB, in the presence of Dy^3+^ and Tb^3+^. The ideal correlations for both FKBP12 and FRB are indicated in lines. The theoretical PCS values were calculated based on the Δχ-tensor parameters for the L3- and L4-FKBP12-rapamycin (Table 2).

Bugayevskiy LM, Snyder JP (1995) Map Projections: A Reference Manual. London/Bristol. Taylor & Francis
