# Supplementary figures and images for: Convenient method for resolving degeneracies due to symmetry of the magnetic susceptibility tensor and its application to pseudo contact shift-based protein–protein complex structure determination
Source: J Biomol NMR. 2012 Apr 10;53(1):53–63. doi: 10.1007/s10858-012-9623-8 (PMC3351616; doi:10.1007/s10858-012-9623-8)

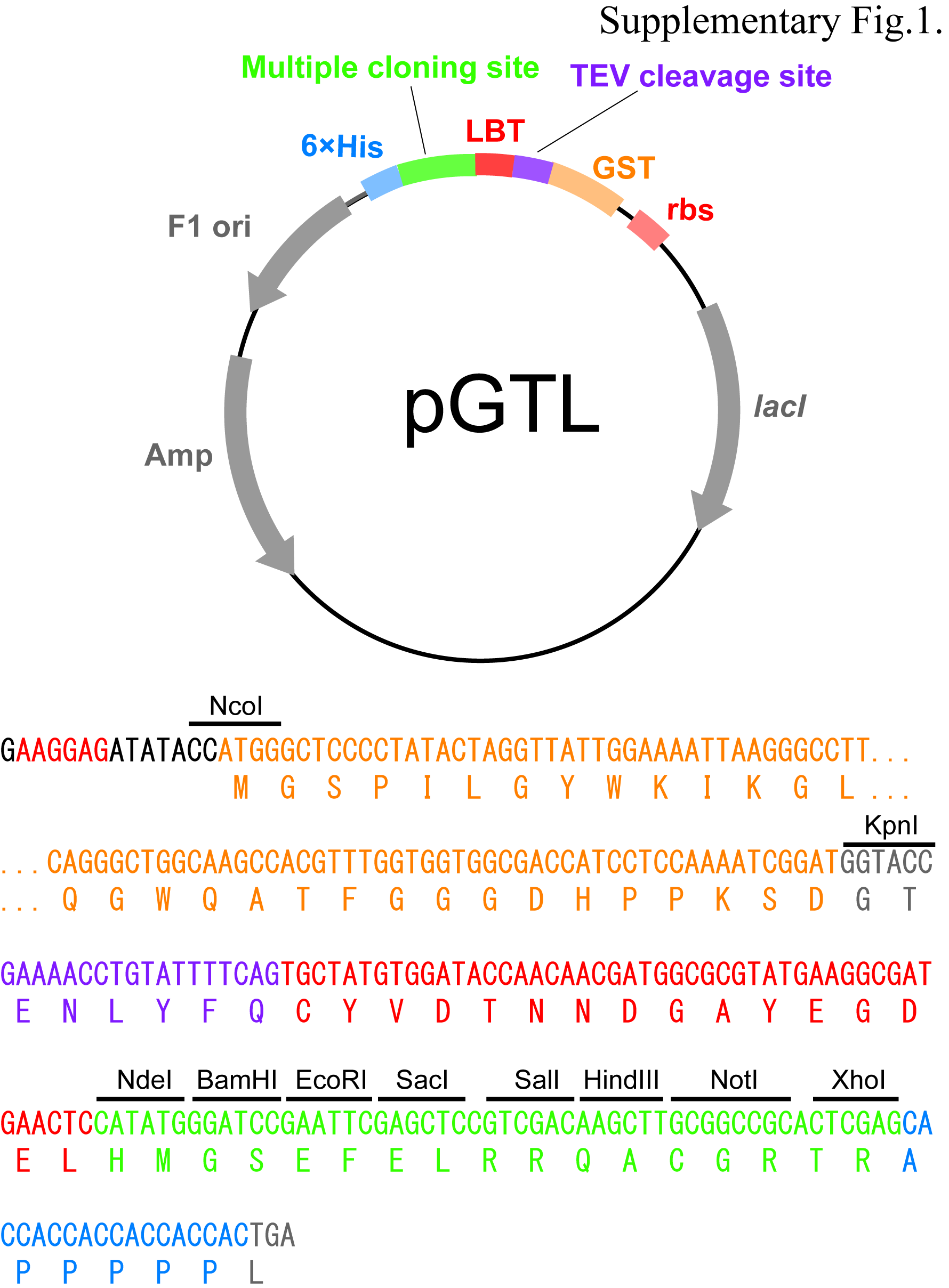

Supplement: Supplementary file 3 — Supplementary material 3 (TIFF 667 kb) [file 10858_2012_9623_MOESM3_ESM.tif]

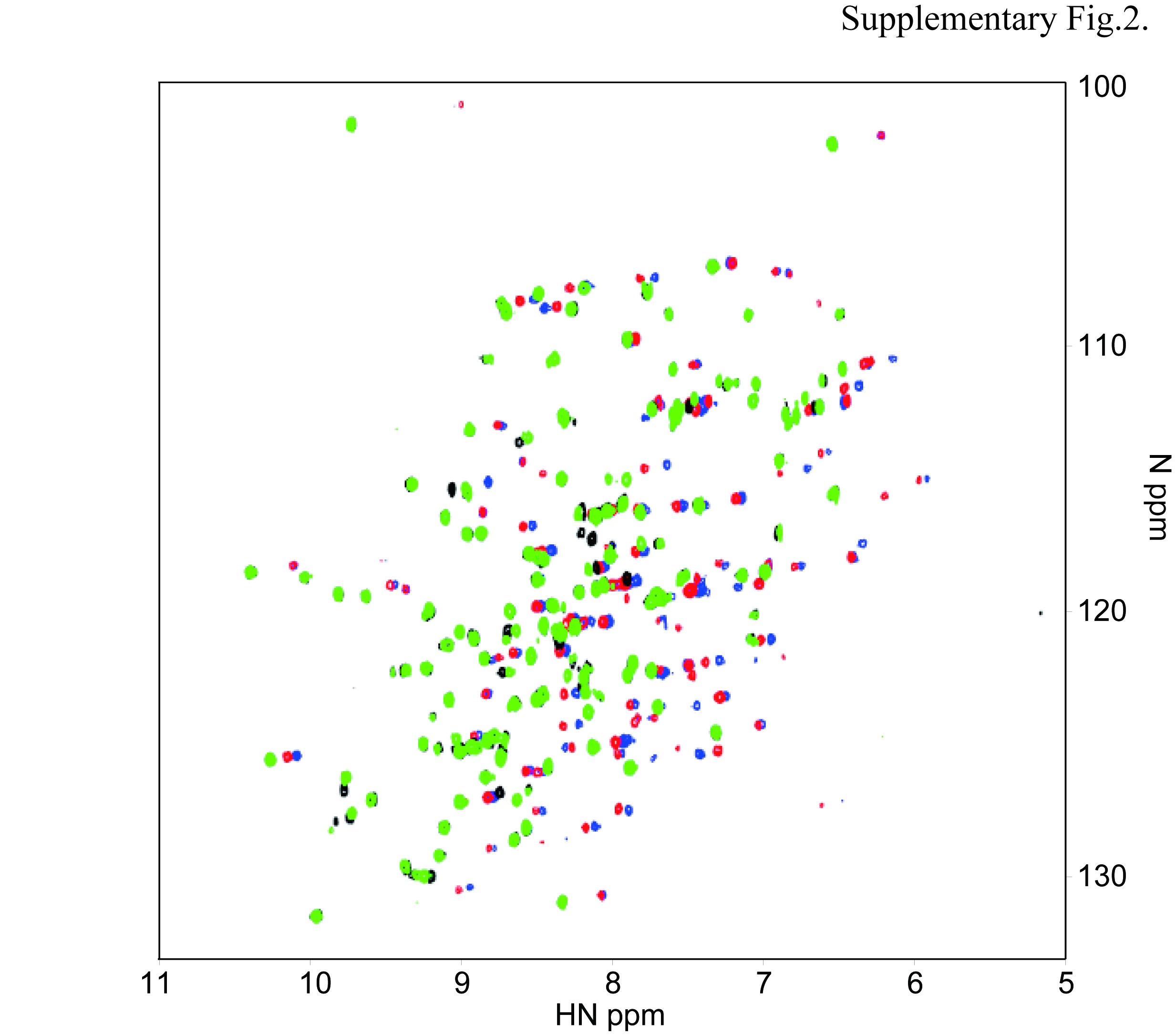

Supplement: Supplementary file 4 — Supplementary material 4 (TIFF 1428 kb) [file 10858_2012_9623_MOESM4_ESM.tif]

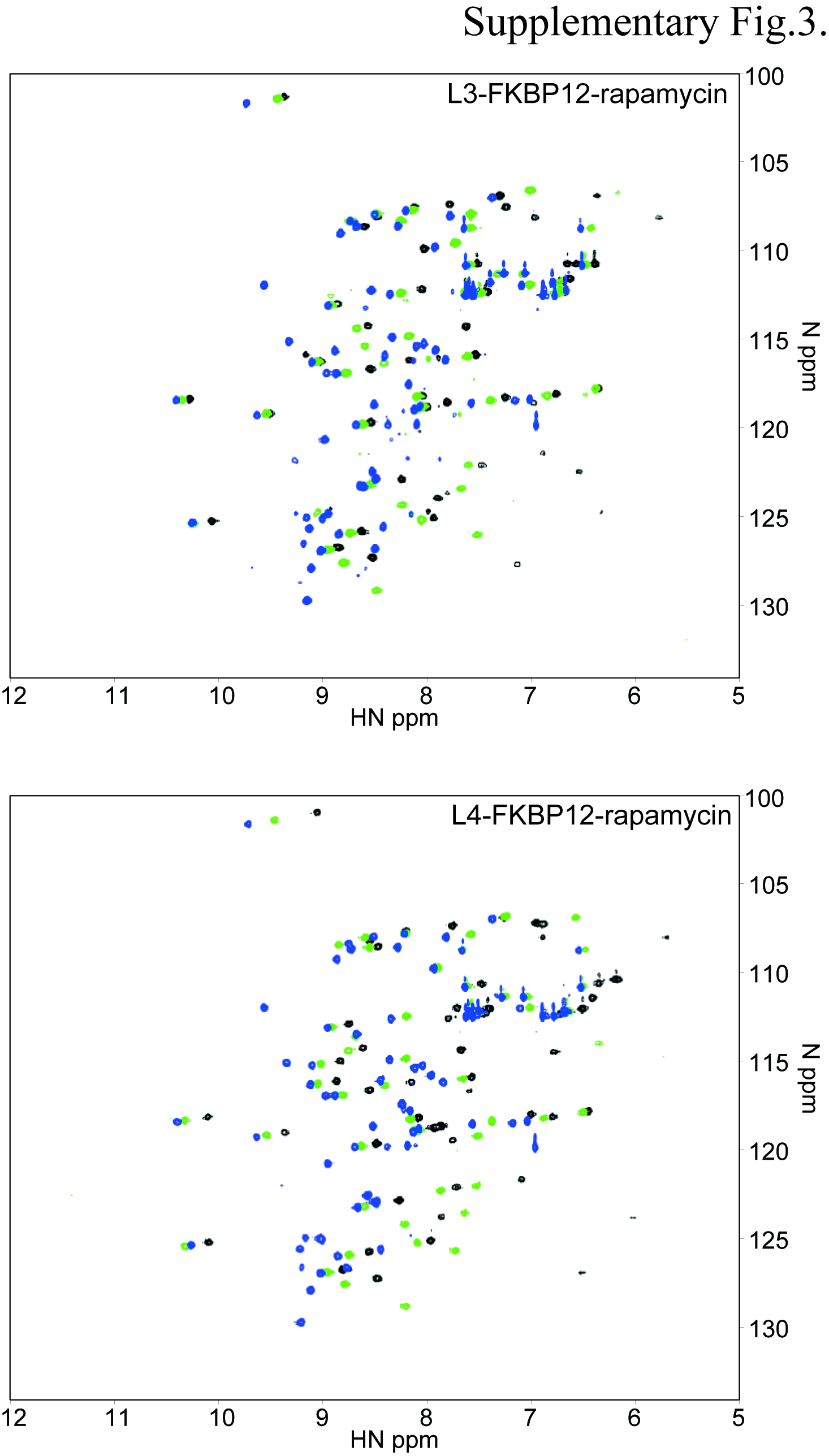

Supplement: Supplementary file 5 — Supplementary material 5 (TIFF 1248 kb) [file 10858_2012_9623_MOESM5_ESM.tif]

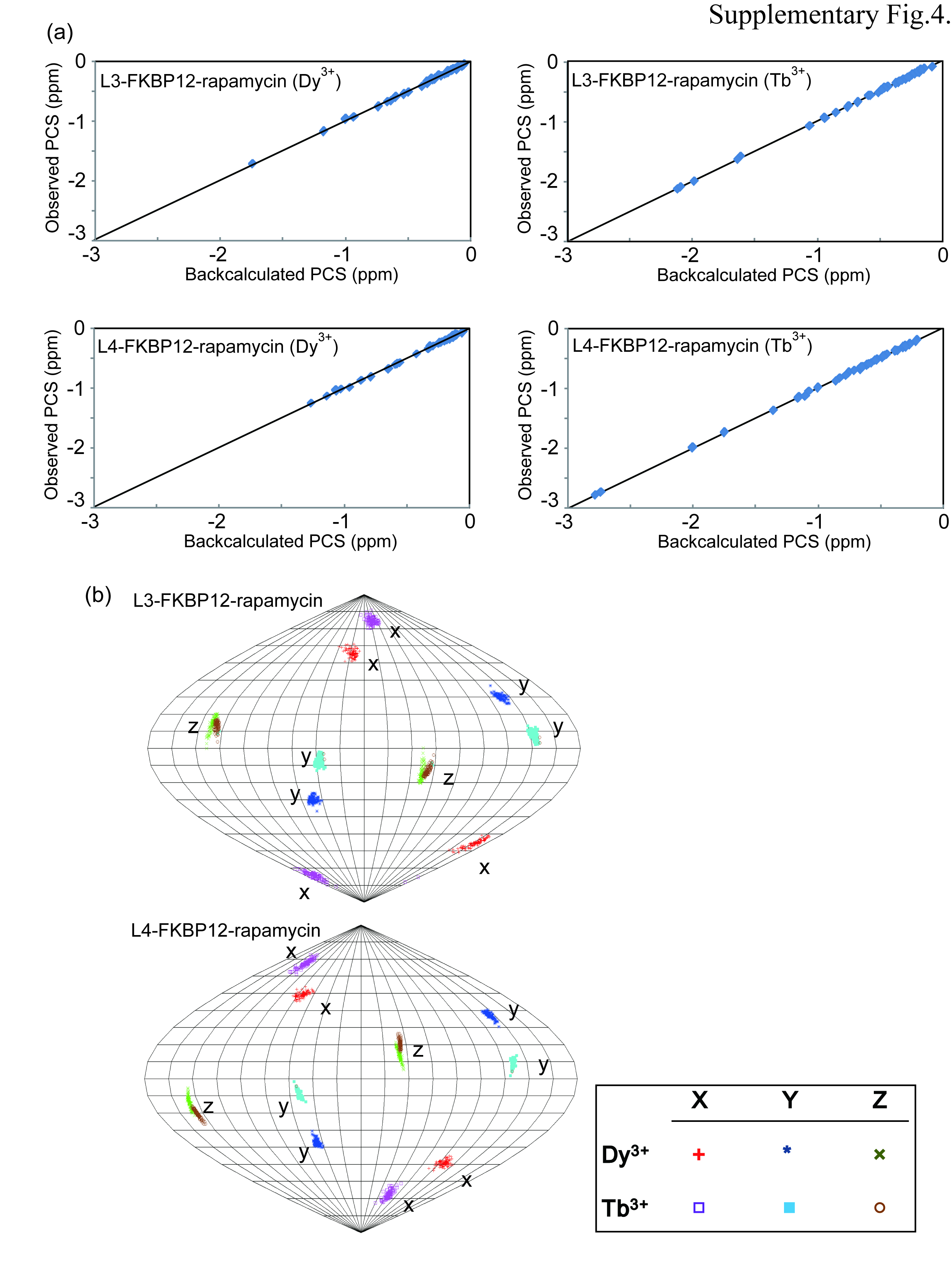

Supplement: Supplementary file 6 — Supplementary material 6 (TIFF 1677 kb) [file 10858_2012_9623_MOESM6_ESM.tif]

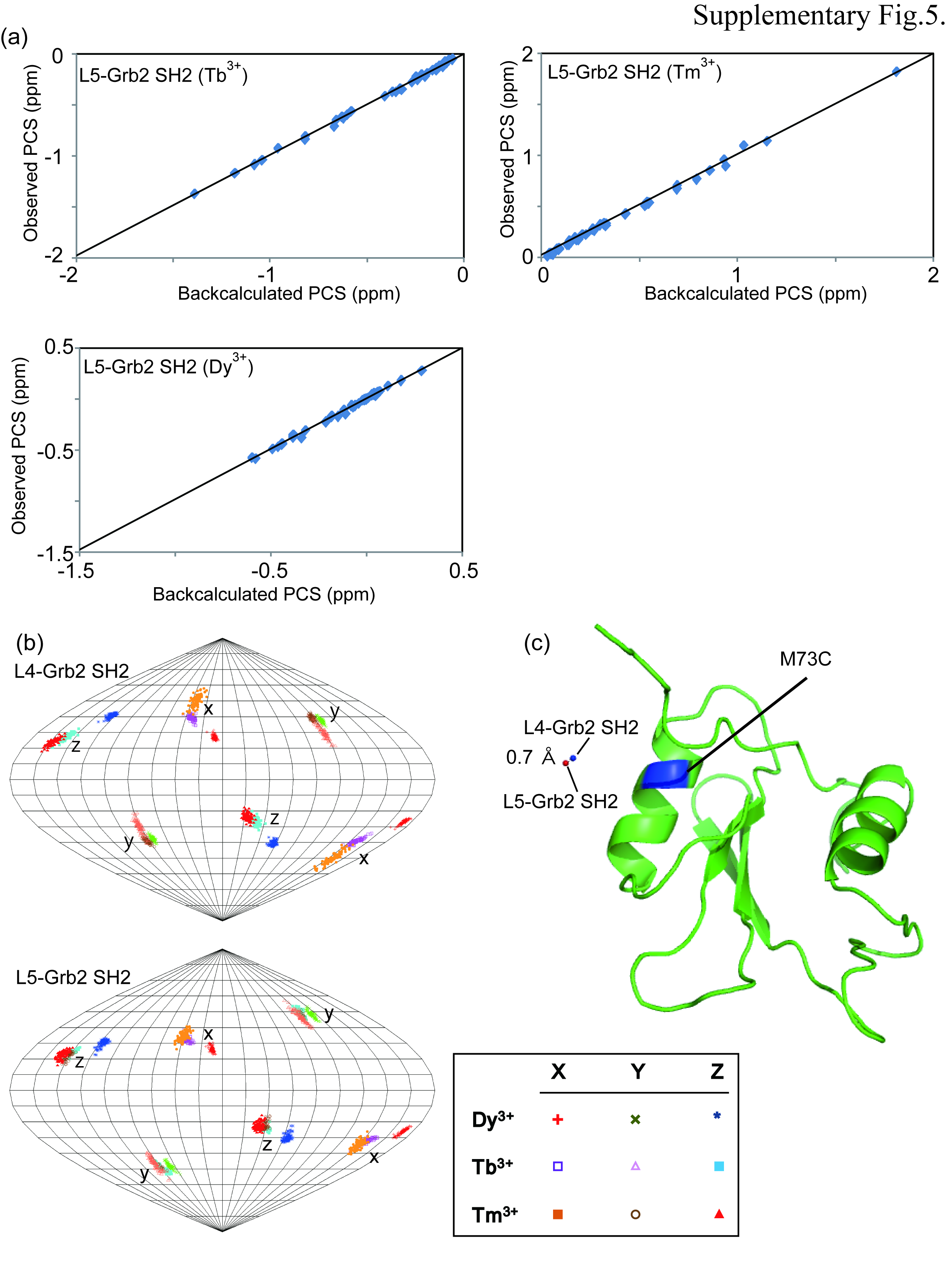

Supplement: Supplementary file 7 — Supplementary material 7 (TIFF 2180 kb) [file 10858_2012_9623_MOESM7_ESM.tif]

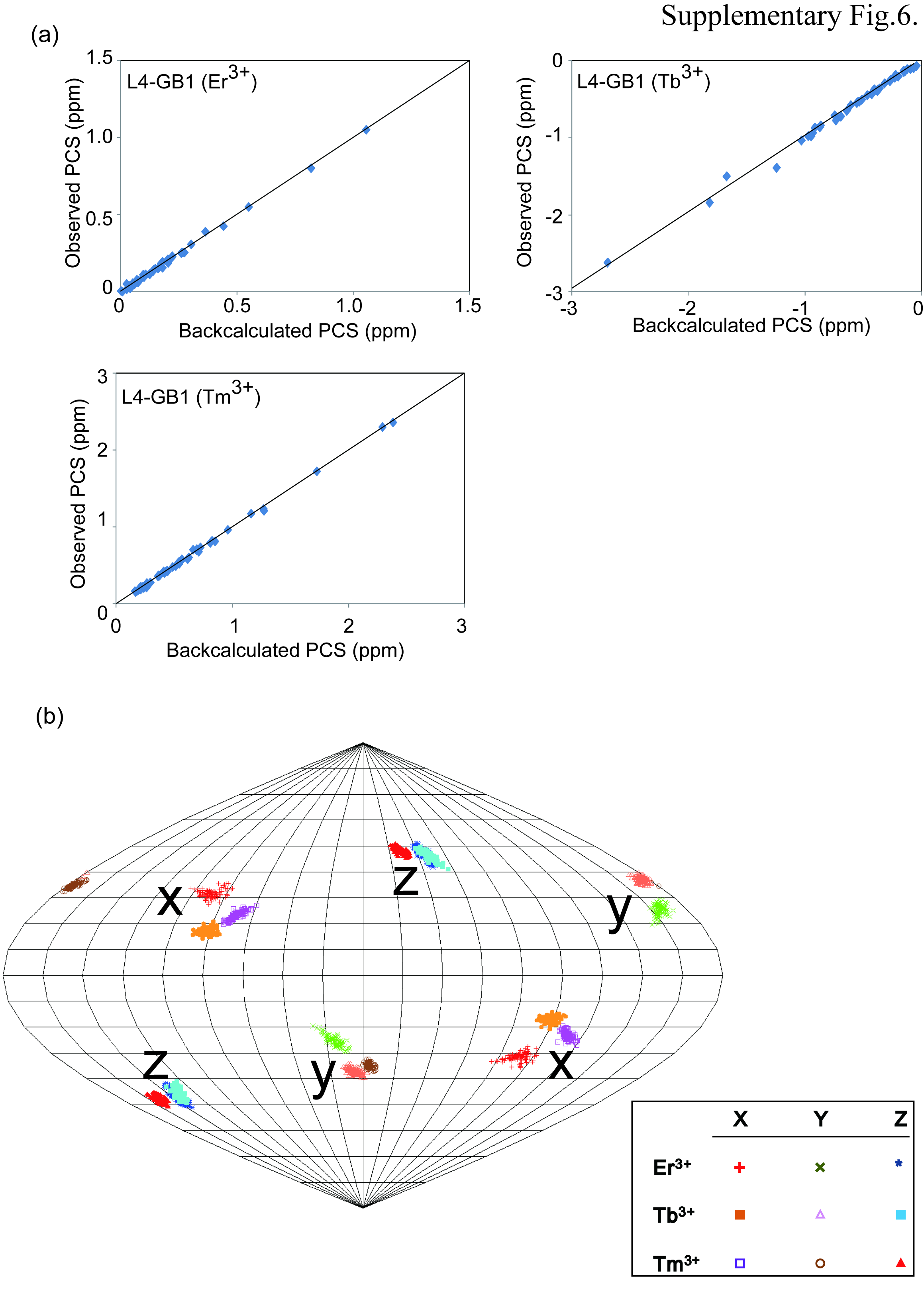

Supplement: Supplementary file 8 — Supplementary material 8 (TIFF 1521 kb) [file 10858_2012_9623_MOESM8_ESM.tif]

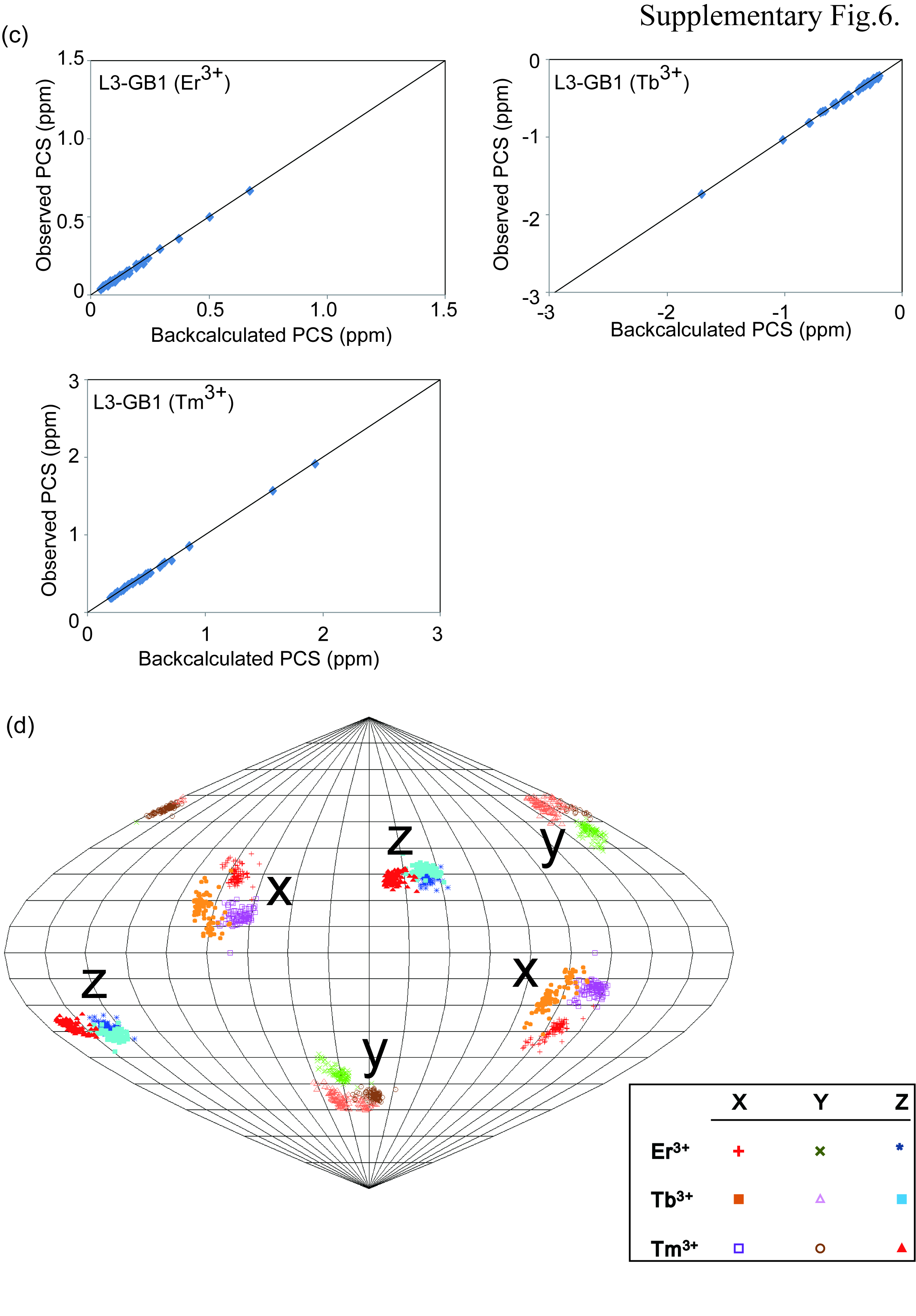

Supplement: Supplementary file 9 — Supplementary material 9 (TIFF 1533 kb) [file 10858_2012_9623_MOESM9_ESM.tif]

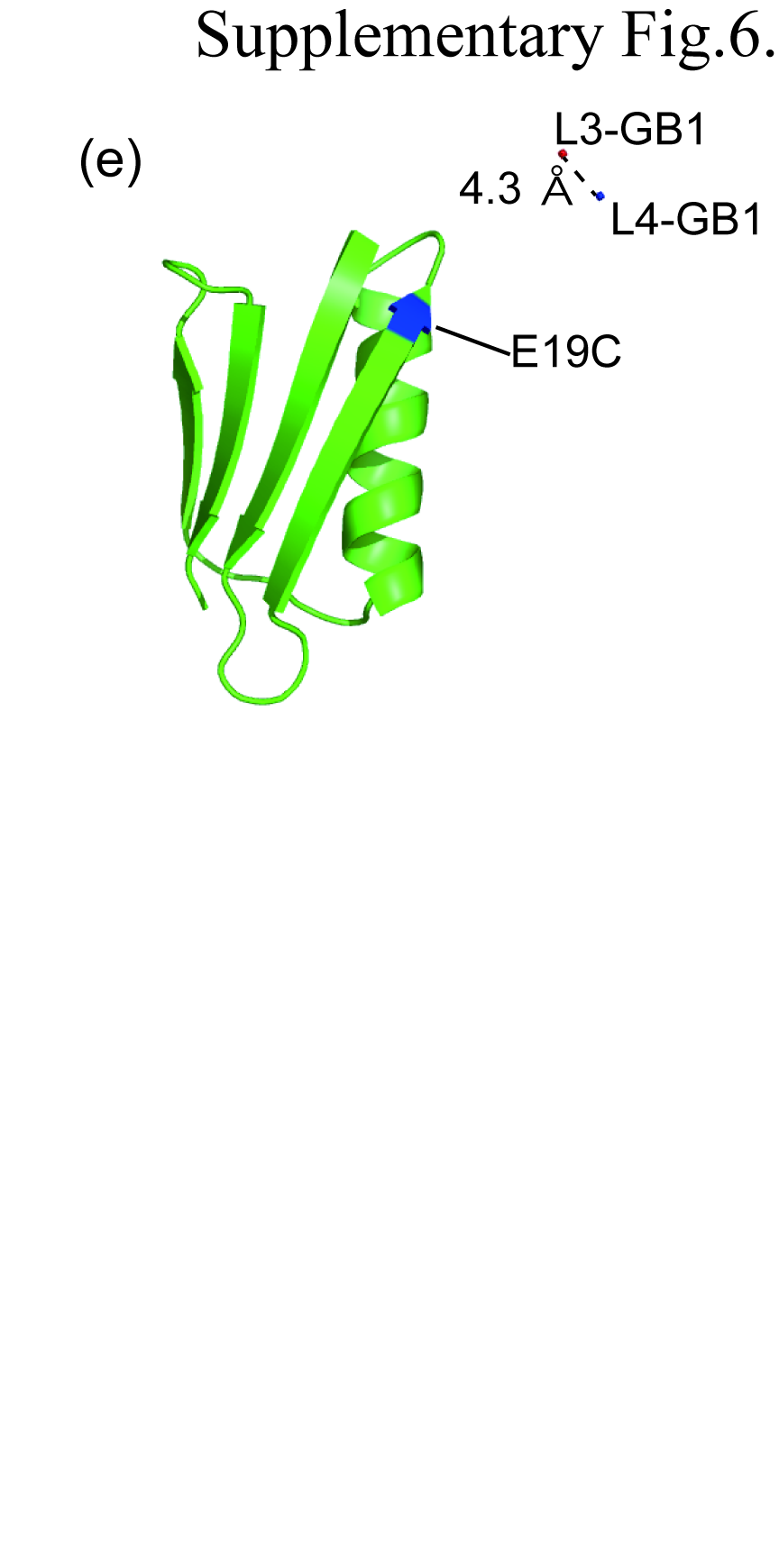

Supplement: Supplementary file 10 — Supplementary material 10 (TIFF 892 kb) [file 10858_2012_9623_MOESM10_ESM.tif]

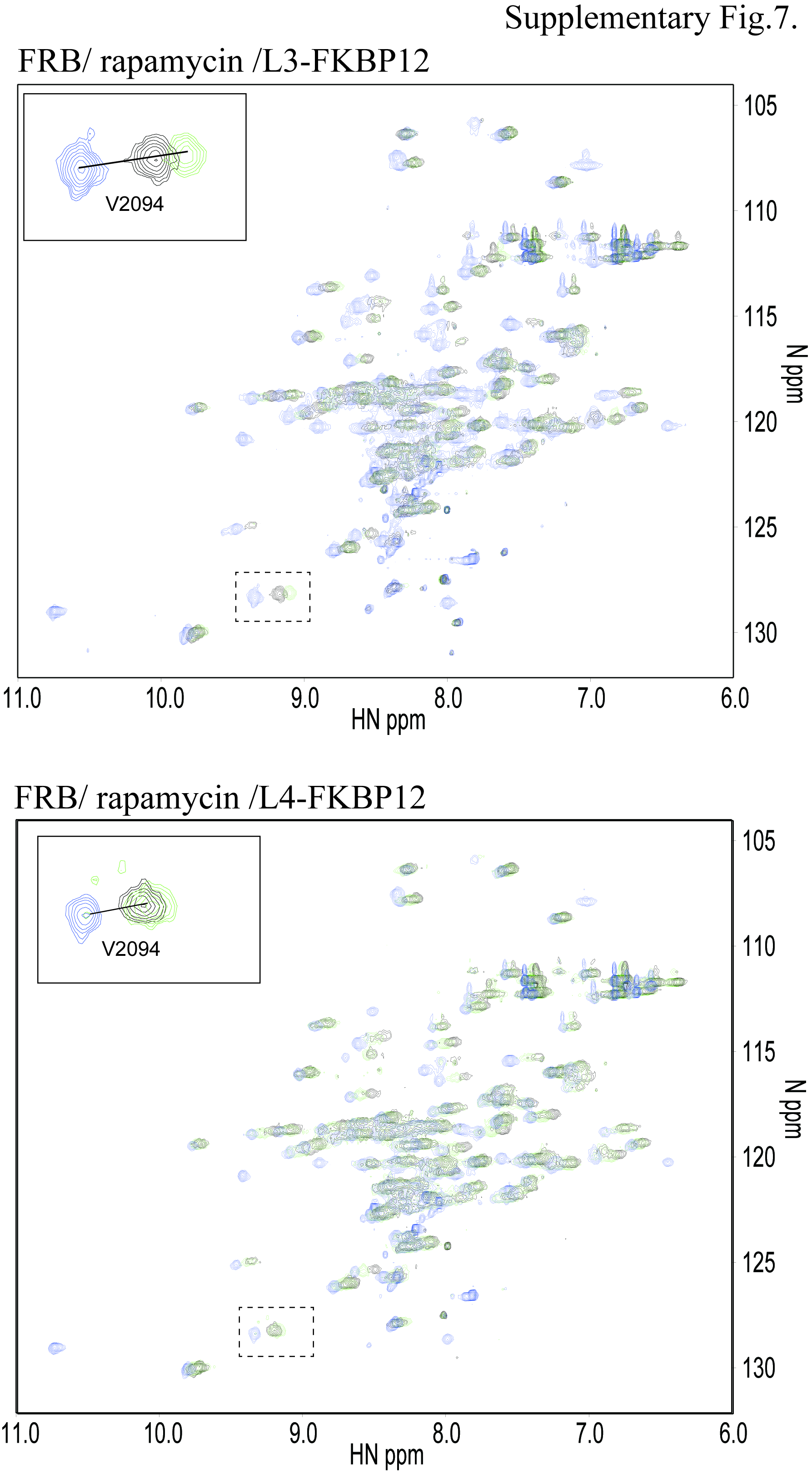

Supplement: Supplementary file 11 — Supplementary material 11 (TIFF 1884 kb) [file 10858_2012_9623_MOESM11_ESM.tif]

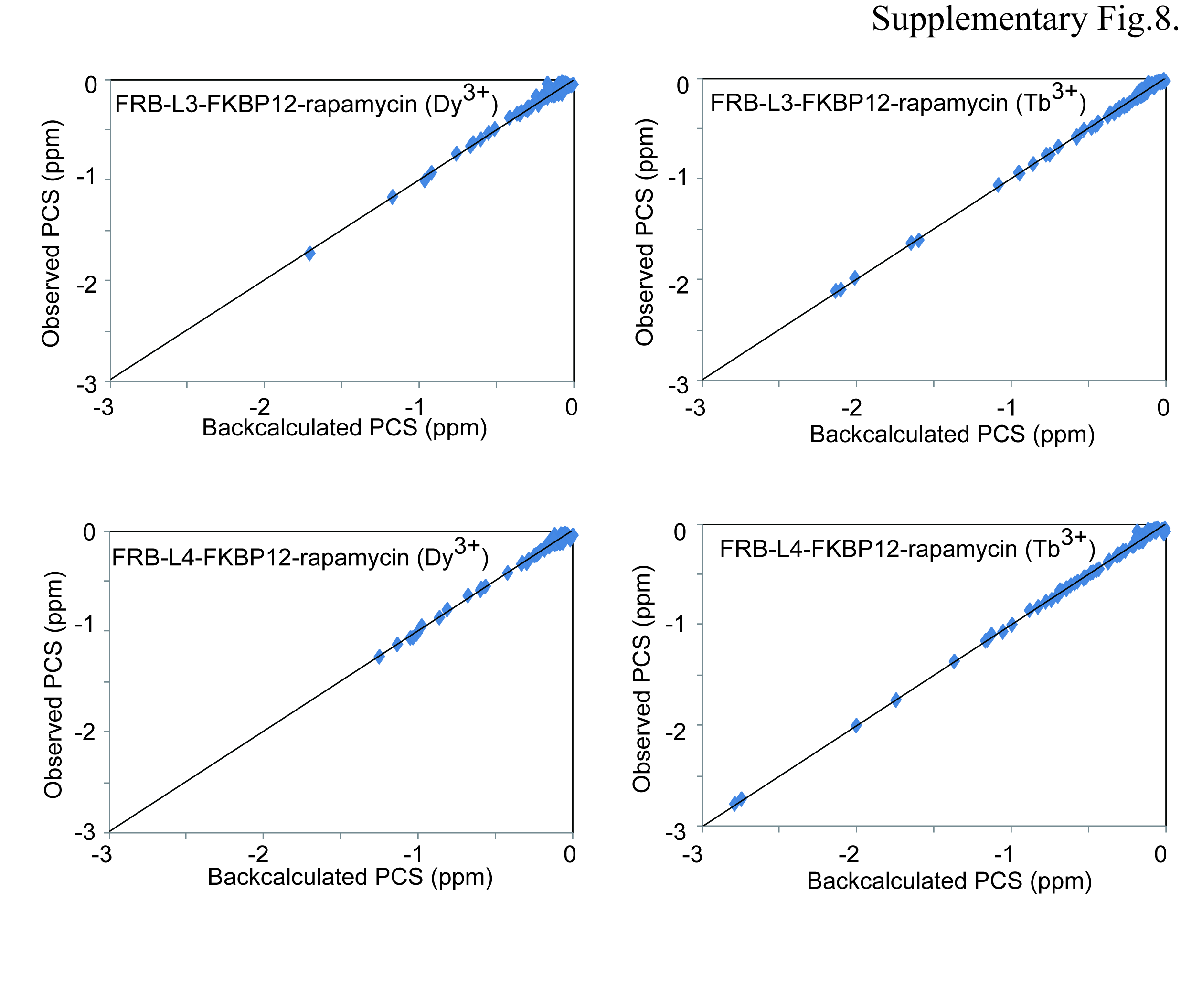

Supplement: Supplementary file 12 — Supplementary material 12 (TIFF 1213 kb) [file 10858_2012_9623_MOESM12_ESM.tif]
