# Supplementary material for: Convenient method for resolving degeneracies due to symmetry of the magnetic susceptibility tensor and its application to pseudo contact shift-based protein–protein complex structure determination
Source: J Biomol NMR. 2012 Apr 10;53(1):53–63. doi: 10.1007/s10858-012-9623-8 (PMC3351616; doi:10.1007/s10858-012-9623-8)
Supplement: Supplementary file 13 — Supplementary material 13 (DOCX 29 kb) [file 10858_2012_9623_MOESM13_ESM.docx]

*XPLOR-NIH input file for docking calculation.*

topology

@TOPPAR:topallhdg.pro

@hicB2.top

end

param

@parallhdg_procheck_05.pro

@hicB.par

end

structure

@dy.psf

@fkbp.psf

@frb.psf

@tb.psf

@axis_new_500.psf

@axis_new_600.psf

@axis_new_700.psf

@axis_new_800.psf

end

delete select

((resi 1 and name ht3) or (resi 108 and name ht3) or

((resi 13 or resi 18 or resi 40 or resi 42 or resi 42 or resi 57 or resi 71) and (name he)) or

((resi 125 or resi 131 or resi 149 or resi 165 or resi 175 or resi 198 or resi 199) and (name he)) or

((resi 17 or resi 34 or resi 35 or resi 44 or resi 47 or resi 52 or resi 73 or resi 105) and (name hz3)) or

((resi 134 or resi 155 or resi 176 or resi 179 or resi 184) and (name hz3)) or

((resi 113 or resi 117 or resi 144 or resi 195 or resi 25) and (name hd1)) or

((resi 94 or resi 87) and (name he2)))

end

evaluate ($knoe = 0.01) !0.01) ! noes

evaluate ($kcdi = 10.0) ! torsion angles

evaluate ($krama = 0.002) !rama

evaluate ($kandb = 0.001)

evaluate ($kimdb = 0.001)

evaluate ($klr = 0.001) !long range rama

evaluate ($kvirt1doverall=0.0)

evaluate ($kvirt1d=0.001)

evaluate ($kvirt2d=0.001)

evaluate ($kvirt3d=0.001)

evaluate ($k_ncs = 200.01)

! Read experimental restraints

noe

nres=30000

class tensor

@tensors.tbl

end

noe

ceiling=10

averaging * cent

potential * soft

scale tensor 500.

scale others 50.

sqoffset * 0.0

sqconstant * 1.0

sqexponent * 2

soexponent * 1

asymptote * 0.1

rswitch * 0.5

end

end

flags exclude * include bonds angle impr vdw noe xpcs xrdc xccr xang end

vector do (fbeta=10) (all)

vector do (mass=100) (all)

xpcs

nres=2000

class terbium3

force 0.8

coeff 9024 3546

@L3TbFrb.tbl

@L3TbFkbp.tbl

class holmium4

force 0.8

coeff 7743 4759

@L4TbFkbp.tbl

@L4TbFrb.tbl

class dysprosium3

force 0.8

coeff 6119 5368

@L3DyFkbp.tbl

@L3DyFrb.tbl

class erbium4

force 0.8

coeff 6130 5050

@L4DyFkbp.tbl

@L4DyFrb.tbl

end

evaluate ($rcon = 0.003)

parameter

nbonds

repel=1.0

rexp=2

irexp=2

rcon=$rcon

nbxmod=3

wmin=0.01

cutnb=4.5 ctonnb=2.99 ctofnb=3.

tolerance=0.5

end

end

set abort off end

constraints inter (resid 1:107) (resid 108:201) end

coordinates @axis_xyzo_3_500.pdb

coordinates @axis_xyzo_3_600.pdb

coordinates @axis_xyzo_3_700.pdb

coordinates @axis_xyzo_3_800.pdb

evaluate ($end_count=10)

evaluate ($count = 0)

while ($count < $end_count ) loop main

evaluate ($count=$count+1)

evaluate ($nodenum=$count+genval)

evaluate ($med="med_str/med_"+encode($nodenum)+".pdb")

coordinates @@$med

!!=== minimization ===!!

evaluate ($cool_steps = 3000)

evaluate ($init_t = 3000.01)

evaluate ($ini_rad = 1.0) evaluate ($fin_rad = 0.78)

evaluate ($ini_con= 0.003) evaluate ($fin_con= 0.004)

evaluate ($ini_ang = 1.0) evaluate ($fin_ang = 1.0)

evaluate ($ini_imp = 1.0) evaluate ($fin_imp = 1.0)

evaluate ($ini_noe = 0.01) !0.01

evaluate ($fin_noe = 0.01)

evaluate ($knoe = $ini_noe) ! slope of NOE potential

flags exclude * include bonds angle impr vdw noe xpcs xrdc xccr xang end

parameters

nbonds

atom

nbxmod 3

wmin = 0.01 ! warning off

cutnb = 4.5 ! nonbonded cutoff

tolerance 0.5

repel= 1.0 ! scale factor for vdW radii = 1 ( L-J radii)

rexp = 2 ! exponents in (r^irex - R0^irex)^rexp

irex = 2

rcon = 0.003 ! actually set the vdW weight

end

end

dynamics internal

reset

itype=powell

stepsize = 0.1

nstep=1000

depred=1

fix = (resi 1:107)

fix = (resi 900)

fix = (resi 901)

group = (resi 108:201)

group = (resi 500)

group = (resi 600)

group = (resi 700)

group = (resi 800)

etol = 0.0000001

gtol = 0.0000001

nprint= 1

end

evaluate ($final_t = 100) { K }

evaluate ($tempstep = 200) { K }

evaluate ($ncycle = ($init_t-$final_t)/$tempstep)

evaluate ($nstep = int($cool_steps*1.6/$ncycle))

evaluate ($bath = $init_t)

evaluate ($k_vdw = $ini_con)

evaluate ($k_vdwfact = ($fin_con/$ini_con)^(1/$ncycle))

evaluate ($radius= $ini_rad)

evaluate ($radfact = ($fin_rad/$ini_rad)^(1/$ncycle))

evaluate ($k_ang = $ini_ang)

evaluate ($ang_fac = ($fin_ang/$ini_ang)^(1/$ncycle))

evaluate ($k_imp = $ini_imp)

evaluate ($imp_fac = ($fin_imp/$ini_imp)^(1/$ncycle))

evaluate ($noe_fac = ($fin_noe/$ini_noe)^(1/$ncycle))

evaluate ($knoe = $ini_noe)

flags exclude * include bonds angle impr vdw noe xpcs xrdc xccr xang end

evaluate ($i_cool = 0)

while ($i_cool < $ncycle) loop cool

evaluate ($i_cool=$i_cool+1)

evaluate ($bath = $bath - $tempstep)

evaluate ($k_vdw=min($fin_con,$k_vdw*$k_vdwfact))

evaluate ($radius=max($fin_rad,$radius*$radfact))

evaluate ($k_ang = $k_ang*$ang_fac)

evaluate ($k_imp = $k_imp*$imp_fac)

evaluate ($knoe = $knoe*$noe_fac)

parameter

nbonds

cutnb=4.5 rcon=$k_vdw nbxmod=3 repel=$radius

end end

dynamics internal

itype=powell

nstep=1000

depred=1

end

end loop cool

dynamics internal

itype=powell

nstep=1000

depred=1

end

flags exclude * include bonds angle impr vdw noe xpcs xrdc xccr xang end

dynamics internal

itype=powell

nstep=1000

depred=1

end

flags exclude * include bonds angle impr vdw noe xpcs xrdc xccr xang end

parameter nbonds rcon 0.004 repel 0.78 end end

dynamics internal

itype=powell

nstep=1000

depred=1

end

flags exclude * include bonds angle impr vdw noe xpcs xrdc xccr xang end

dynamics internal

itype=powell

nstep=1000

depred=1

end

flags exclude * include bonds angle impr vdw noe xpcs xrdc xccr xang end

parameter nbonds rcon 0.004 repel 0.78 end end

dynamics internal

itype=powell

nstep=1000

depred=1

end

!!=== Write out the final structure ===!!

print threshold=0.5 noe

evaluate ($rms_noe=$result)

evaluate ($violations_noe=$violations)

xrdc print threshold 0.10 all end

evaluate ($rms_xrdc=$result)

evaluate ($violations_xrdc=$violations)

print thres=0.05 bonds

evaluate ($rms_bonds=$result)

print thres=5. angles

evaluate ($rms_angles=$result)

print thres=5. impropers

evaluate ($rms_impropers=$result)

remarks ============================================================

remarks overall,bonds,angles,improper,vdw,noe

remarks totalE : $ener, $bond, $angl, $impr, $vdw, $noe

remarks

remarks xpcs,xrdc,xccr,xang

remarks energies: $xpcs, $xrdc, $xccr, $xang

remarks ============================================================

remarks bonds,angles,impropers,noe

remarks rms-d: $rms_bonds,$rms_angles,$rms_impropers,$rms_noe

remarks

remarks xpcs,xrdc,xccr,xang

remarks rms-d: $rms_xpcs,$rms_xrdc,$rms_xccr,$rms_xang

remarks ============================================================

remarks noe

remarks violations.: $violations_noe

remarks

remarks

remarks xpcs,xrdc,xccr

remarks violations.: $violations_xpcs,$violations_xrdc,$violations_xccr

remarks xang

remarks violations.: $violations_xang

remarks ============================================================

evaluate ($filename = "FINAL/comp_" + encode($nodenum) + ".pdb")

write coordinates output=$filename end

end loop main

stop
